# Functional similarity, despite taxonomical divergence in the millipede gut microbiota, points to a common trophic strategy

**DOI:** 10.1186/s40168-023-01731-7

**Published:** 2024-01-29

**Authors:** Julius Eyiuche Nweze, Vladimír Šustr, Andreas Brune, Roey Angel

**Affiliations:** 1 Institute of Soil Biology and Biogeochemistry, Biology Centre CAS, České Budějovice, Czechia; 2 Faculty of Science, University of South Bohemia, České Budějovice, Czechia; 3 RG Insect Gut Microbiology and Symbiosis, Max Planck Institute for Terrestrial Microbiology, Marburg, Germany

**Keywords:** Polysaccharide degradation, Hindgut microbiota, Millipede holobiont, Symbiosis, Glycoside hydrolases, Nutrient cycling, Acetogens, Ecosystem engineers

## Abstract

**Background:**

Many arthropods rely on their gut microbiome to digest plant material, which is often low in nitrogen but high in complex polysaccharides. Detritivores, such as millipedes, live on a particularly poor diet, but the identity and nutritional contribution of their microbiome are largely unknown. In this study, the hindgut microbiota of the tropical millipede *Epibolus pulchripes* (large, methane emitting) and the temperate millipede *Glomeris connexa* (small, non-methane emitting), fed on an identical diet, were studied using comparative metagenomics and metatranscriptomics.

**Results:**

The results showed that the microbial load in *E. pulchripes* is much higher and more diverse than in *G. connexa*. The microbial communities of the two species differed significantly, with *Bacteroidota* dominating the hindguts of *E. pulchripes* and *Proteobacteria* (*Pseudomonadota*) in *G. connexa*. Despite equal sequencing effort, de novo assembly and binning recovered 282 metagenome-assembled genomes (MAGs) from *E. pulchripes* and 33 from *G. connexa*, including 90 novel bacterial taxa (81 in *E. pulchripes* and 9 in *G. connexa*). However, despite this taxonomic divergence, most of the functions, including carbohydrate hydrolysis, sulfate reduction, and nitrogen cycling, were common to the two species. Members of the *Bacteroidota* (*Bacteroidetes*) were the primary agents of complex carbon degradation in *E. pulchripes*, while members of *Proteobacteria* dominated in *G. connexa*. Members of *Desulfobacterota* were the potential sulfate-reducing bacteria in *E. pulchripes*. The capacity for dissimilatory nitrate reduction was found in *Actinobacteriota* (*E. pulchripes*) and *Proteobacteria* (both species), but only *Proteobacteria* possessed the capacity for denitrification (both species). In contrast, some functions were only found in *E. pulchripes*. These include reductive acetogenesis, found in members of *Desulfobacterota* and *Firmicutes* (*Bacillota*) in *E. pulchripes*. Also, diazotrophs were only found in *E. pulchripes*, with a few members of the *Firmicutes* and *Proteobacteria* expressing the *nifH* gene. Interestingly, fungal-cell-wall-degrading glycoside hydrolases (GHs) were among the most abundant carbohydrate-active enzymes (CAZymes) expressed in both millipede species, suggesting that fungal biomass plays an important role in the millipede diet.

**Conclusions:**

Overall, these results provide detailed insights into the genomic capabilities of the microbial community in the hindgut of millipedes and shed light on the ecophysiology of these essential detritivores.

Video Abstract

**Supplementary Information:**

The online version contains supplementary material available at 10.1186/s40168-023-01731-7.

## Introduction

Plant litter is the primary source of food and shelter for detritivorous animals [1], of which millipedes are one of the largest and most diverse members [2]. However, detritivores generally lack enzymes to digest complex polysaccharides [3, 4], which make up most of the plant litter biomass [5]. Instead, many rely on their gut microbiome to break down various hydrocarbon substrates [6] and release simple sugars or short-chain fatty acids that the host can absorb [7, 8]. In arthropods, the gut microbiome plays an important role in the development and adaptation of the host to its trophic niche [9–11]. Like all other soil arthropods, millipedes (class: Diplopoda) host a diverse community of microorganisms in their guts, which may be essential to the host’s nutrition [12, 13]. In millipedes, the midgut and hindgut compartments are colonized by a dense population of aerobic and anaerobic bacteria, with the highest microbial density found in the hindgut [12].

Unlike the microbiome of other important detritivores, primarily termites [14] and earthworms [15], the millipede microbiome has received little attention so far. Only a handful of prokaryotic surveys were conducted, mostly using basic culture-dependent and molecular fingerprinting techniques [16]. Since many host-associated microorganisms cannot be grown outside their hosts, our knowledge remains limited. Recent studies have reported the most prevalent taxa in the millipede species *Anadenobolus monilicornis* [12] (only a preprint of a metagenomic study is available) and *Telodeinopus aoutii* [17] (only transcriptome data).

Freshly fallen leaf litter or wood bark contains mainly pectin, starch, cellulose, hemicellulose, and lignin [5]. The latter three are insoluble and chemically recalcitrant due to their dense structure [18] and are typically only hydrolyzed by microorganisms [19]. Indeed, gut extracts and even cultivated aerobes from several millipedes were shown to hydrolyze cellulose, hemicelluloses, and pectin [20–23]. These reports were supported by a recent metatranscriptomic study, where bacteria were shown to be the primary producers of hydrolytic enzymes in the tropical millipede *Telodeinopus aoutii* [24].

However, whether millipedes—like termites [25]—benefit directly from the lignocellulolytic activity of their gut microbiota or even rely on it as a primary source of nutrition remains an open question. Several researchers have hypothesized in the past that millipedes ingest litter primarily as a means of providing a substrate for microorganisms (bacteria, fungi, and lichens), which in turn serve as their food source [26, 27]. Accordingly, the central role of the millipede is to mix the litter layers, mechanically fragment the plant material, and inoculate the pieces with gut bacteria and fungi. If correct, we expect to see an expression of glucanase and chitinase genes related to fungal cell wall degradation [28].

Despite the progress made in understanding the ecophysiology of the millipede holobiont, it remains unclear whether millipedes rely on fermentative degradation of cellulose to generate volatile fatty acids for their nutrition. Despite their common detritivorous lifestyle, some species were shown to be CH_4_ emitters, while others were not, which has been attributed to differences in size and the resulting redox conditions in their digestive tracts [29, 30]. Since CH_4_ is an end product of the cellulose degradation cascade under anaerobic conditions, a lack of methane production could indicate differences in the underlying microbial fermentations.

In addition to providing the enzymes required for the digestion of lignocellulose, millipede gut bacteria may also play other nutritional roles, such as fixing nitrogen and recycling nutrients, that compensate for their nitrogen-poor diets [31, 32]. However, it is unknown if millipedes can fix and recycle nitrogen, and a comprehensive molecular approach is needed to provide answers to these questions.

In this study, we used metagenomic and metatranscriptomic sequencing of the gut microbiome of two millipede model species to shed light on their metabolic potential and better understand the trophic niche of these keystone detritivores. We analyzed individuals of lab-maintained *Epibolus pulchripes* (order: Spirobolida) and *Glomeris connexa* (order: Glomerida). Both feed on senescent leaves but differ in size and habitat. *E. pulchripes* is a fairly large (130–160 mm) tropical millipede, widely spread along the East African coast [33], which has been shown to be a strong methane emitter [29]. *G. connexa* is a small (10–17 mm) species common to Central Europe [34] that was shown to be a non-methane emitter [30]. Our analysis covered genes involved in carbon, sulfur, and nitrogen cycling. In particular, we focused on carbohydrate-active enzymes (CAZymes) with secretion signal peptides targeting substrates from plant, fungi, and microbial origin.

## Methods

### Millipede sources and rearing conditions

Juvenile individuals of the tropical millipede *Epibolus pulchripes* were obtained from a breeding colony maintained in our lab. The animals are kept in a plastic terrarium (60 × 30 × 20 cm) on a forest floor substrate with peat, rotten wood, and a blend of leaf litter from maple, oak, Canadian poplar, and beech trees. The environment was maintained at 25 ℃ and subjected to a 12-h photoperiod under controlled conditions. Moisture was maintained by regularly spraying with tap water. The temperate *Glomeris connexa* was collected from a forest near the Helfenburk castle near Bavorov (49° 8′ 10.32′′ N, 14° 0′ 24.21′′ E) in the Czech Republic. The collection of this species required no special permission. Both millipedes were identified down to the species level based on morphological features ([35, 36]; data not shown).

The animals were maintained in the laboratory for 25 days before dissection. Both species were kept in the lab in plastic terraria with aeration holes. The boxes contained commercial fine sand and *Populus x canadensis* (Canadian poplar) leaf litter. High humidity was maintained by spraying with tap water every other day. Both species were kept at near-optimal temperatures: *E. pulchripes* was kept at 25 oC in a light-regulated room, with a maximum of one individual in a box (19.3 × 13.8 × 5 cm). For *G. connexa*, five individuals were kept in a box (15 × 10 × 4 cm) at 15 °C in an incubator.

### Acetylene reduction assay

ARA was performed as previously described [37] by placing a single millipede in 100 ml Schott DURAN borosilicate glass bottles, with or without leaf litter and supplementing the headspace with 4% acetylene (final conc.). Ethylene accumulation was measured at 0, 4, and 6 h by directly injecting 500 µl headspace gas into a GC (HP 5890 Series II equipped with a Porapak N column and an FID detector, Hewlett Packard).

### Bacterial counts

Three pellets of fresh feces were collected from the millipede boxes at once using sterilized tweezers, suspended in 1 ml of phosphate buffer (pH 7.4) and plated 20 µl in triplicates on Lysogeny broth (LB) agar, and incubated at 25 °C. After 16 h, the colonies from each pellet were counted.

### Nucleic acid extraction

Three replicates were analyzed for each millipede species. Because of the difference in body size, a single individual of *E. pulchripes* and five of *G. connexa* (from the same rearing box) were considered technical replicates. Animals were dissected, according to Sardar et al. [17]. The intact hindguts were separated and stored at − 20 °C until nucleic acid extraction. The total nucleic acids (TNA) were extracted from the hindguts and feces, purified, and quantified according to Angel et al. [38]. Briefly, each sample (0.677–1.108 g for *E. pulchripes* and 0.083–0.092 g for *G. connexa*) was subjected to 3-consecutive bead beating rounds (Lysing Matrix E tubes; MP Biomedicals™) in a FastPrep-24™ 5G (MP Biomedicals™) in the presence of CTAB, phosphate buffer (pH 8.0), and phenol. The extract was then purified using phenol–chloroform-isoamyl alcohol (25:24:1; Thermo Scientific™), precipitated using a PEG solution with Invitrogen™ UltraPure™ Glycogen (Thermo Fisher Scientific) as a co-precipitant and purified using OneStep™ PCR Inhibitor Removal Kit (Zymo Research). The complete protocol is available online [39]. The quantity and quality of the DNA were determined using the Quant-iT™ PicoGreen HS Assay Kit (Thermo Fisher Scientific™) and the Agilent 2100 Bioanalyzer (Agilent). RNA was purified from the TNA extracts using TURBO™ DNase and the GeneJET RNA Cleanup and Concentration Micro Kit (Thermo Fisher Scientific). The RNA was quantified using the Quant-it RiboGreen RNA Assay Kit (Thermo Fisher Scientific). The quality of the RNA was evaluated by Novogene Sequencing – Europe (Cambridge, UK) using agarose gel electrophoresis and Agilent 2100 Bioanalyzer.

### Amplicon library preparation, gene quantification, and sequencing

The bacterial diversity in the hindgut compartments from the two millipede species was analyzed by paired-end sequencing of the V4 region of the 16S rRNA genes on an Illumina MiniSeq platform (2 × 250 cycle configuration; V2 reagent kit; Illumina) at the DNA Services Facility at the University of Illinois, Chicago, USA (Table S1), following Naqib et al. [40]. After quantifying the DNA with PicoGreen, the samples were diluted to a final concentration of 10 ng µl^−1^. For PCR and library preparation, the primers 515F_mod and 806R_mod [41] were used to amplify the V4 region of the 16S rRNA gene. For gene quantification, the template DNA was diluted to 0.01 ng µl^−1^, and 2 µl was used per reaction with primers 338F–805R (0.5 µM), the 516P FAM/BHQ1 probe (0.2 µM) together with the digital droplet PCR Supermix for probes (Bio-Rad), and quantified on a QX200 AutoDG Droplet Digital PCR System (ddPCR; Bio-Rad). The full protocol can be found online [42]. The copy numbers of 16S rRNA were normalized for 1 ng of total DNA.

### Library preparation and sequencing for metagenome and metatranscriptome

Library preparations, sequencing of the metagenomes and metatranscriptomes (see below), and quality control were provided by Novogene (UK) Company Limited. Metagenomic libraries were prepared using the same DNA preparations described above. Sequencing libraries were generated using NEBNext® Ultra™ DNA Library Prep Kit by Illumina (NEB, USA) following the manufacturer’s recommendations, and index codes were added to attribute sequences to each sample. The libraries were pooled and sequenced on an Illumina NovaSeq PE 150 platform, generating an average of 50.3 G for *E. pulchripes* and 41.3 G base pairs for *G. connexa*.

Metatranscriptomic libraries were prepared using quality-controlled RNA preparations at the Novogene (UK) Company Limited. The RNA was sequenced on an Illumina NovaSeq PE150 platform and generated 321.8 G reads. Briefly, three sample quality control methods were used: nanodrop, Agarose Gel Electrophoresis, and Agilent Bioanalyzer 2100. The rRNA was depleted using the Ribo-Zero kit (Thermo Fisher Scientific). Sequencing libraries were generated using the NEBNext® UltraTM RNA Library Prep Kit for Illumina® (NEB, Ipswich, MA, USA) following the manufacturer’s instructions, and index codes were added to assign sequences to specific samples. The quality control for the library preparation included quantification and integrity evaluation using Qubit 2.0 (Thermo), Agilent 2100 Bioanalyzer System (Agilent Technologies), and qPCR to exclude DNA contamination.

### Reconstruction of metagenome-assembled genomes

The raw sequence reads (from the metagenome and metatranscriptome libraries) were quality-filtered using Trimmomatic v0.39 [43]. Quality-filtered metagenomic reads from both millipede species were uploaded into anvi’o v7 metagenomic workflow [44], co-assembled (de novo) with MEGAHIT v1.2.9 [45], and assembled contigs < 1 kbp were removed. Bowtie2 V2.3.4.3 [46] was used for mapping the quality trimmed reads to the initial co-assembled contigs (before removing potential eukaryotic contigs) and SAMtools v2.4.2 [47] to sort the output SAM files into BAM files. We used the anvi-display-contigs-stats function to get a summary of contigs statistics from the co-assembly of each millipede separately and both species together. The open reading frames were identified with Prodigal v2.6.3 [48] and single-copy core genes (SCG) with HMMER v3.3.2 [49]. The gene-level taxonomy was predicted using Centrifuge v1.03-beta [50] and annotated functions using the NCBI’s Clusters of Orthologous Groups (COG) [51] and KEGG Orthologs (KOs) databases [52]. Both Metabat2 v2.12.1 [53] and CONCOCT V0.38 [54] were used to create contigs clusters (bins) and the anvi’o interactive interface to refine the bins manually. Comparing the two methods, Metabat2 yielded higher quality MAGs, while many CONCOCT MAGs suffered from high contamination levels and taxonomic misclassification (data not shown). Therefore, only the Metabat2 MAGs were kept for downstream analysis. We retained all prokaryotic metagenome-assembled genomes (MAGs) with more than 50% completion and redundancy in SCG below 10% based on CheckM [55]. The anvi-gen-phylogenomic-tree function was used to plot a phylogenomic tree by concatenating 39 single-copy genes from Bacteria_71 (ribosomal proteins) from the recovered MAGs.

### Taxonomic classification of sequence data

Unless mentioned otherwise, all data processing steps and plotting were done in R [56]. After amplicon sequencing, the 16S rRNA reads were demultiplexed using cutadapt V3.5 [57]. The raw reads were processed, assembled, and filtered using DADA2 v1.26, with the standard filtering parameters, according to Callahan [58]. Unique sequences were identified and clustered into amplicon sequence variants (ASV). Chimaeras were removed with the removeBimeraDenovo function. The quality-filtered pair-end reads were classified to the genus level using the Genome Taxonomy Database (GTDB) [59]. The resulting tables were merged into a Phyloseq object [60]. Decontamination was done using decontam v1.18 [61]. After rarefying the dataset without replacement, we calculated the Principal Coordinates Analysis (PCoA) using the unweighted UniFrac as distance [62].

To profile the prokaryotic community in the metagenome, the quality-filtered metagenomic reads from each millipede species were again co-assembled (de novo) with MEGAHIT v1.2.9 [45], and assembled contigs < 1 kbp were removed. For metatranscriptome, remnant rRNA reads were removed using SortMeRNA v4.3.4 [63], and the resulting non-rRNA reads for each millipede species were co-assembled (de novo) using Trinity v2.13.2 [64]. Next, all contigs sized < 500 bp were discarded. Potential eukaryotic contigs from both library types were removed using Whokaryote [65], which does not consider contigs with less than two genes. The prokaryotic contigs were taxonomically classified using the Contig Annotation Tool (CAT) v5.2.3 [66] based on the GTDB. Unclassified clades and clades with < 200 contigs at the phylum level were ignored*.* The taxonomy of the MAGs was inferred using GTDB-Tk v2.0.0 [67], which uses a 95% ANI cutoff for the species boundary to determine the phylogenetic placement and relative evolutionary divergence (RED) values of query genomes in the GTDB reference tree [58]. Genomes were defined as novel genera (all MAGs clustered at 60% AAI [68] without a genus GTDB-Tk assignment), novel species (GTDB-Tk ANI output < 95%), and novel strains (GTDB-Tk ANI output < 99%) [69]. The eukaryotic community structure in the metagenomes was determined using METAXA2 [70], extracting SSU and LSU rRNA sequences. These were attributed to various origins. Non-bacterial rRNA sequences were validated via a blast analysis [71]. Metatranscriptomic reads were taxonomically classified using local blastn against the nodes.dmp and names.dmp database files.

### Functional annotation

The prokaryotic co-assembled reads from metagenome and MAGs were profiled for functional traits and metabolism using the METABOLIC v4.0 pipeline with default parameters [72]. We predicted the genes for carbohydrate-active enzymes (CAZymes), such as glycoside hydrolases (GHs), carbohydrate-binding modules (CBMs), polysaccharide lyases (PLs), and carbohydrate esterases (CEs), based on the dbCAN2 meta server. Along with this, signal peptide predictions were based on the SignalP 4.1 databases [73]. Further screening of CAZyme genes was performed manually, and CAZymes were defined as those predicted by at least two tools. Lastly, the putative substrates for the glycoside hydrolases were predicted based on information from the literature.

The gene homologs for acetogenesis, hydrogenases, nitrogen, and sulphur cycling were also predicted. Six genes for reductive acetogenesis absent in the pipeline were annotated with blastp [74] at an *e* value of 1e − 30. Blastp [71] was also used to confirm all the predictions from METABOLIC (maximum of five target sequences). The resulting data were imported and plotted in the R packages ggplot2 [75], circlize [76], and iTOL [77].

### Relative abundance of MAGs and gene families

To determine the relative abundance of the MAGs in both metagenome and metatranscriptome, we mapped the reads from both library types to the MAGs. Each MAG’s sample-specific mean coverage was used to calculate its relative abundance using CoverM v0.6.1 (https://github.com/wwood/CoverM) with default parameters of the coverm-genome function. CoverM used Minimap2 [78] for mapping and calculating read coverage per genome (relative abundance) with a –min-read-percent-identity of 90%.

For the abundance of the gene families in both metagenome and metatranscriptome, the reads from both library types were mapped to each gene, and the mean coverage was used to estimate the relative abundance in Transcripts per million (TPM) using CoverM within contig with –min-read-percent-identity of 90% to allow comparing the datasets. CoverM used the bwa-mem aligner [79]. TPM in gut metagenomes reflects the relative abundance of a gene in the bacterial community. Genes with a zero TPM value were removed, and the values were converted to log(TPM + 1) for plotting. The quantification of SSU/LSU rRNA sequences in the metagenome and eukaryotic contigs in the metatranscriptome followed a similar methodology.

## Results

### Bacterial load and 16S rRNA gene diversity in the millipede guts

**Fig. 1:**
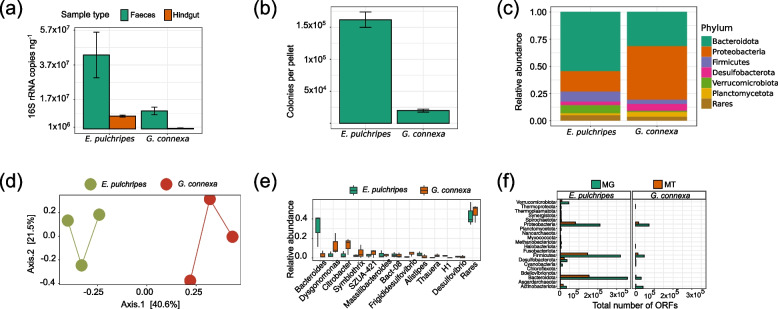
Microbial load and community composition of the gut microbiome in *Epibolus pulchripes* and *Glomeris connexa*. **a** 16S rRNA gene copies in the hindgut and faecal samples from *E*. *pulchripes* and *G*. *connexa*. **b** Average colony counts from faeces samples grown on LB agar media. **c** Relative abundance of bacteria from the hindgut at phylum level based on Illumina sequencing of the 16S rRNA gene. **d** PCoA-analysis based on unweighted UniFrac distances between the microbial communities in the hindguts of both millipede species. **e** The relative abundance of dominant bacteria at the genus level. **f** Taxonomic classification of the prokaryotic community in the assembled metagenomes (MG) and metatranscriptomes (MT) from hindgut samples of *E*. pulchripes and *G*. connexa based on the total ORFs

Quantification of the 16S rRNA gene copies in the hindgut using ddPCR yielded 0.74 × 10^7^ in *E. pulchripes* and 0.39 × 10^7^ per ng DNA in *G. connexa* (Fig. 1a; Table S1). The respective microbial load in the feces was 6 and 26 times higher than in the hindguts. Comparing these numbers with the number of viable bacteria in the feces (using a number of colonies per fecal pellet) revealed similar differences in microbial load and that a large proportion of the bacteria remained uncultured (Fig. 1b, Table S1).

The amplicon sequencing of the V4 region of the bacterial and archaeal 16S rRNA gene amplified from the millipede hindguts yielded an average of 38,317 high-quality reads. Of the 16 phyla represented in the dataset, the majority of sequences in *E. pulchripes* and *G. connexa* datasets were *Bacteroidota* (54.6% and 31.6%) and *Proteobacteria* (*Pseudomonadota*; 18.8% and 49.2%), followed by *Verrucomicrobiota* (7.6% and 1.1%), *Firmicutes* (*Bacillota*; 9.4% and 4.0%), *Desulfobacteriota* (3.1% and 6.4%), and *Plancomycetota* (1.8% and 4.1%; Fig. 1c, Table S2). However, despite the many shared phyla, the communities differed significantly on the genus level, with only 14.24% shared between the species (Fig. 1d). Following the trends on the phylum level, the two species differed in the relative abundance of these common genera. In *E. pulchripes*, the community was highly dominated by *Bacteroides* (*Bacteroidaceae*, 31.1%), followed by more minor members such as *Alistipes* (*Rikenellaceae*, 3.7%), *Massilibacteroides* (*Tannerellaceae*, 3.3%), *Dysgonomonas* (*Dysgonomonadaceae*, 3.1%), and others (Fig. 1e). In contrast, the distribution of genera in *G. connexa* was shallower and dominated by *Dysogonomonas* (12.9%) and *Citrobacter* (12.6%), and others.

### Quality of metagenome and metatranscriptome assemblies

The six metagenomic libraries from the two millipede species yielded 0.63 G paired-end reads (Table S3). *E. pulchripes* samples were separately assembled with Megahit into 823.6 K contigs (total length – 2.9 Gb). The reads from *G. connexa* were assembled into 162.8 K contigs (total length – 0.5 Gb). Meanwhile, the metatranscriptomes yielded an average of 0.35 G paired-end*. In E. pulchripes*, the reads were assembled into 1.3 M contigs, while In *G. connexa*, the assembled reads constituted 136.0 K contigs (Table S4).

### Microbial abundance in metagenomic and metatranscriptomic reads across hindgut samples

Filtering contigs of eukaryotic origin yielded 338,035 (41%) prokaryotic contigs for *E. pulchripes* and 62,892 (39%) for *G. connexa* (Table S5). For both species, 95% of the metagenomic contigs could be taxonomically assigned. In contrast, the metatranscriptomes contained only 17% and 7% of prokaryotes that also passed size and quality filtering. Of those, 58% in *E. pulchripes* and 74% in *G. connexa* could be taxonomically assigned, at least at the phylum level. The taxonomic classification of the contigs resembled the composition obtained from the amplicon sequencing, except for *Firmicutes*, which were under-represented in our amplicon library compared to the metagenome and metatranscriptome. Namely, in *E. pulchripes*, *Bacteroidota* was the most abundant and active member of the community, with 34% of the total ORFs in both metagenomes and metatranscriptomes (Fig. 1e), followed by *Firmicutes* (30.3% and 32.1%), *Proteobacteria* (20% and 18%), *Verrucomicrobiota* (4.3%, 1.9%), *Desulfobacterota* (3.4% and 4.7%), and *Actinobacteriota* (3.1% and 3.6%). In samples from *G. connexa*, *Proteobacteria* was the highest-ranked taxon with 71,847 ORFs (37.1%) and 16,777 (47.1%) in metagenomes and metatranscriptomes, followed by *Firmicutes* (24% and 36%), *Actinobacteriota* (21% and 6.4%), *Bacteroidota* (16% and 7%), and *Desulfobacterota* (1% and 2.1%). In terms of non-bacterial diversity, as expected, methanogenic *Euryarchaeota* (mainly orders *Methanobacteriales*, *Methanomassiliicoccales*, and *Methanosarcinales*) were detected in *E. pulchripes*, but also some in the non-CH_4_-emitting in *G. connexa* (Table S5). As for fungi, the phylum *Ascomycota* was found to be the most abundant among eukaryotes (> 90% of the fungal contigs) in both millipede species. In addition, *E. pulchripes* also hosted Nematoda (42.8%) and *Ciliophora* (12.6%) in large numbers, while *in G. connexa*, *Apicomplexa* (74.2%), and *Metamonada* (18.2%) were the most dominant (Fig. S1; Table S5). The protist order *Eccrinales*, typically found microscopically in millipedes, was only represented by a single (*E. pulchripes*) or double (*G. connexa*) very rare contigs and was absent in the metatranscriptome (Table S5). The metatranscriptome profiling largely agreed with the metagenome regarding the taxonomic profile, but the relative abundances were significantly different, possibly due to the small size of the eukaryotic dataset (Fig. S1; Table S5).

### De novo assembly of genomes and phylogenomic distribution

**Fig. 2:**
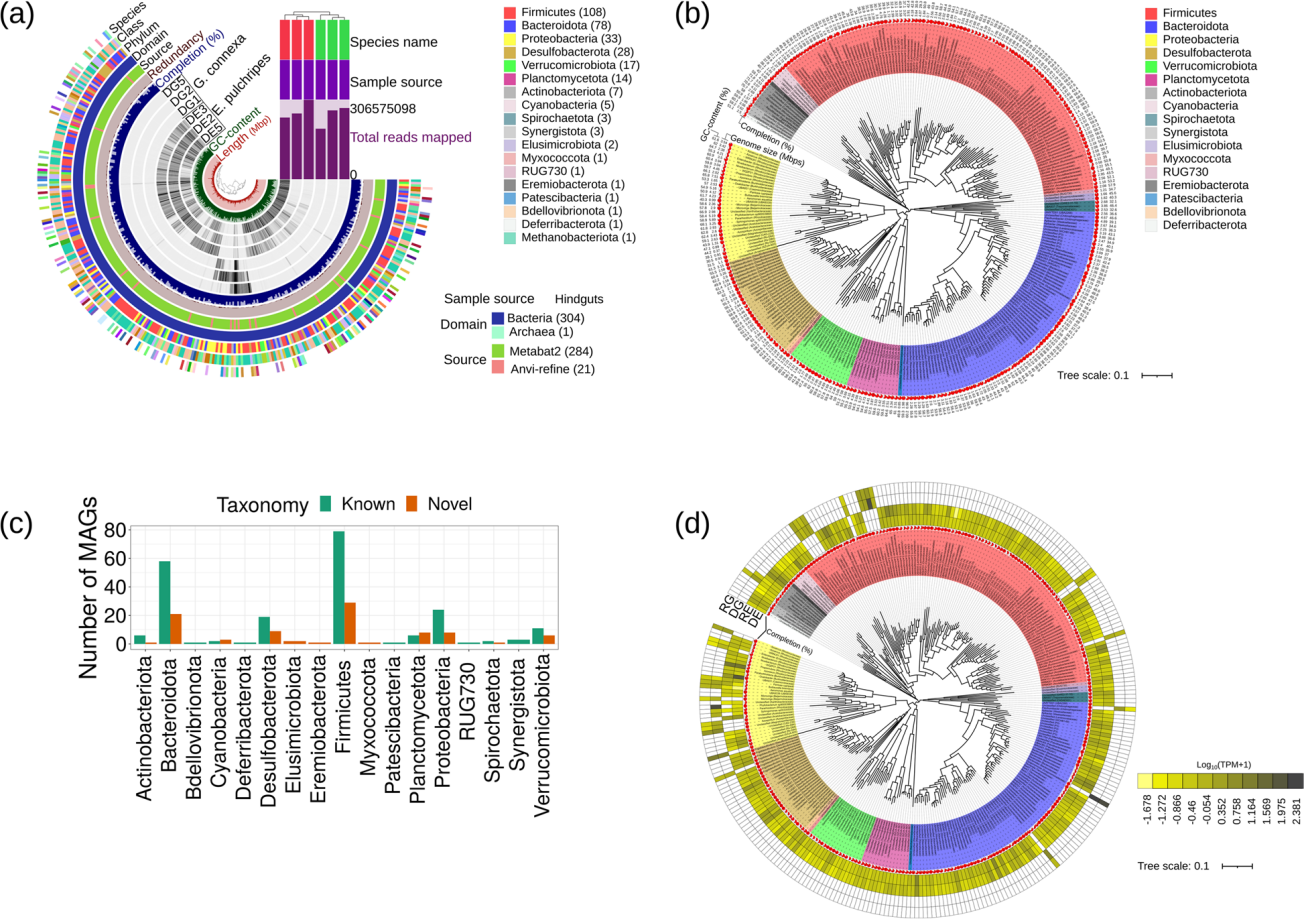
Taxonomic composition of the recovered MAGs. **a** Static images from anvi’o’s interactive display for recovered MAGs from the hindguts of *E*. *pulchripes* (DE2, 3, 5) and *G*. *connexa* (DG1, 2, 5). The tree (dendrogram) at the central section of the anvi’o interactive image shows the hierarchical clustering of MAGs based on their sequence composition and their distribution across samples. From inner to outer layers: length layer (shows the actual length of a genome in Mbps), GC-content, four view layers with information about MAGs across samples (mean coverage), completion, redundancy, source (automatically binned with MetaBAT2 and manually refined), domain of the MAGs (archaea or bacteria), genome phylum, class and species based on GTDB-Tk. The bars show the total number of reads mapped, sample source (hindgut) and sample names (*E*. *pulchripes* and *G*. *connexa*) (**b**) Phylogenomic tree based on 39 concatenated bacterial single copy gene (ribosomal proteins, see Table S8). **c** Potentially novel species from the hindgut of *E*. *pulchripes* and *G*. *connexa* identified with GTDB-Tk based on relative evolutionary distance. **d** The relative abundance of MAGs in the metagenomic and metatranscriptomic read samples, estimated for each sample replicate and the average was used in the plotting. DE and RE indicate the relative abundance of MAGs in the metagenome and metatranscriptomes of *E*. *pulchripes*, whereas DG and RG indicate the relative abundance of MAGs in the metagenome and metatranscriptomes of *G*. *connexa*

The metagenomic reads from both millipede species were co-assembled, binned, and refined, generating 305 MAGs, each with completeness > 50% and redundancy < 8.5% (Fig. 2a, Table S6). Notably, 47% of these MAGs exhibited a completeness of 90% or more, while 62% of the overall MAGs attained a completeness level of 80% or higher. One MAG was assigned to archaea and the rest to bacteria. The genome sizes ranged from 0.36 to 7.76 Mbp. We concatenated the amino acid sequences of the bacterial single-copy core genes from the MAGs (Table S6) and constructed a phylogenomic tree (Fig. 2b). After assigning taxonomy with GTDB-Tk (Tables S7 and S8), 108 MAGs (35.5%) were placed into the phylum *Firmicutes*, including the families of *Lachnospiraceae*, *Ruminococcaceae*, CAG-74 and some unclassified groups. The phylum *Bacteroidota* followed with 79 MAGs (26%), represented mainly by the families of *Tannerellaceae*, *UBA932*, *Bacteroidaceae*, *Azobacteroidaceae*, and *Rikenellaceae*. Thirty-two of the MAGs (10.5%) were placed into the *Proteobacteria*. In particular, we identified the *Alphaproteobacteria*, *Rs-D84*, *Beijerinckiaceae*, *Acetobacteraceae*, and the *Gammaproteobacteria*, *Enterobacteriaceae*, *Rhodocyclaceae*, and *Burkholderiaceae*. Other core phyla were *Desulfobacterota* (28 MAGs, 9.2%), *Verrucomicrobiota* (17 MAGs, 6%), *Planctomycetota* (14 MAGs, 5%), and *Actinobacteriota* (7 MAGs, 2.30%). Notably, ninety-one MAGs representing potentially novel bacterial species (81 in *E. pulchripes* and 10 in *G. connexa*) were identified based on relative evolutionary distance (RED) (Fig. 2c). The novel species belonged mainly to the *Firmicutes* and *Bacteroidota* phyla.

### Distribution of MAGs in metagenomes and metatranscriptomes

Of the retrieved MAGs, 272 (92%) were represented in *E. pulchripes* and only 23 in *G. connexa* (Fig. 2a and d). Ten of the MAGs were present in both species (Table S8). The MAGs were mapped to the quality-filtered metagenomic and non-rRNA pair-end reads to calculate the variation in abundance for each MAG across the samples. Additionally, the number of MAGs in each sample was estimated considering as “absent” those with abundances < 0.001% (Table S9). This analysis revealed that 66–75% and 36–44% of the metagenomic reads remained unmapped in *E. pulchripes* and *G. connexa*. In metatranscriptomes, 71–86% and 82–85% of reads were unmapped in the samples from *E. pulchripes* and *G. connexa*. Two *Proteobacteria* MAGs from *E. pulchripes* and six MAGs from *G. connexa* (3 *Proteobacteria*, 2 *Firmicutes*, and 1 *Actinobacteriota*) remained unmapped in the metatranscriptomic samples.

### The repertoire of bacterial carbohydrate-degrading enzymes

The degradation of plant litter requires the concerted work of carbohydrate-active enzymes (CAZymes), including glycoside hydrolases (GHs), carbohydrate-binding modules (CBMs), carbohydrate esterases (CEs), glycosyltransferases (GTs), polysaccharide lyases (PLs), auxiliary activities (AAs), and S-layer homology modules (SLHs). We analyzed the MAGs for the presence of such CAZymes, focusing on proteins with secretion signal sequences (SSPs), which are secreted or targeted to other locations, such as the periplasmic space or bacterial cytoplasmic membrane [80]. However, we acknowledge that some bacterial proteins have been found to be secreted without any apparent signal peptide [81]. Annotation of the predicted amino acid sequences revealed 24,690 and 2042 CAZymes in the MAGs from *E. pulchripes* and *G. connexa*, respectively. Of these, 7721 and 352 had SSPs (Table S10). Among the potentially secreted CAZymes in *E. pulchripes*, GHs were the most abundant (82.3%), followed by CEs (8.8%), PLs (6.2%), CBMs (3.4%), GTs (1.1%), AAs (0.2%), and SLHs (0.03%). Also in *G. connexa*, GHs were the most abundant (73.6%) among the potentially secreted CAZymes. GHs were also the most abundant of all expressed CAZymes in *E. pulchripes* (80.3% of 6199) and *G. connexa* (73.4% of 215).

GHs (glycoside hydrolases) were classified into 127 families by the CAZyme database, and their substrate specificity can be predicted based on this structure (Table S11). Our annotation results showed that some GHs are located on the same MAGs with one or more CBMs, GTs, CEs, PLs, or other GHs (Table S12), suggesting that the CAZymes involved in polysaccharides degradation are organized in clusters.

**Fig. 3:**
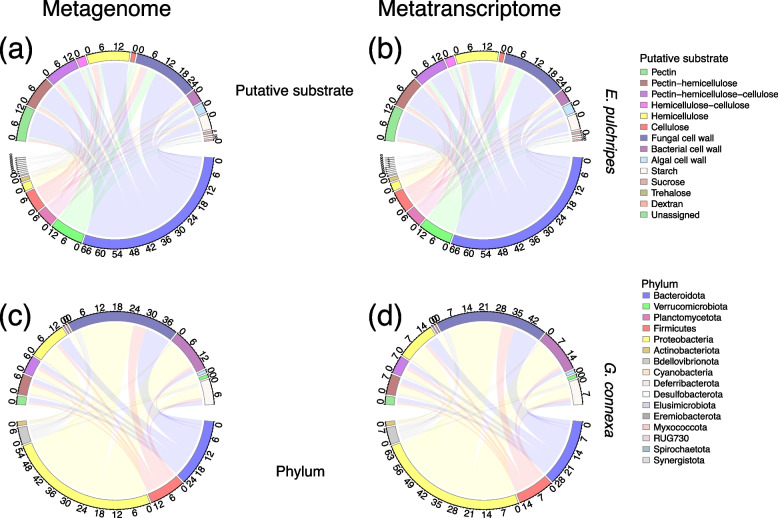
Relative abundance of glycoside hydrolases (GHs) with secretion signal peptides in metagenome-assembled genomes (MAGs) and their corresponding transcripts. The GHs were grouped at the family level and according to their putative substrates (top of the chord) and the taxa contributing to the GHs (bottom of the chord). Chord (**a**) displays the contribution of GHs from different phyla in metagenomes, while chord (**b**) shows its corresponding GH transcripts from the hindgut of *E*. *pulchripes*. Chord (**c**) shows the abundance of GHs at the phylum level in metagenomes, while chord (**d**) displays its corresponding GH transcripts from the hindgut of *G*. *connexa*. The pair-end reads of both library types were mapped to the genes to get the coverage and calculate the relative abundance in transcripts per million (TPM).  The mean TPM was calculated from the three replicate samples and summed for each taxonomic level

In *E. pulchripes*, the majority of secreted GHs (6199) belonged to *Bacteroidota* (64%; presented in TPM), *Verrucomicrobiota* (12.2%), and Firmicutes (9.1%) (Fig. 3a). These same phyla also expressed the highest amount of GHs (6199), with *Bacteroidota* contributing the most (64%; presented in TPM) (Fig. 3b; Table S12). Based on the predicted substrate specificity, the secreted GHs from the MAGs assigned to *Bacteroidota* (4153) had the capability for the degradation of fungal cell walls (25%; presented in TPM), hemicellulose (17%), pectin (16%), pectin-hemicellulose (13.2%), pectin-hemicellulose-cellulose (13%), starch (6.5%), algal cell wall (4.2%), and bacterial cell wall (4.2%). The same pattern was also observed in the expressed GHs, although the relative abundance of the transcripts was lower than those in the metagenomes. *Bacteroidota’s* capabilities were mainly contributed by the families of *Bacteroidaceae* (17%; presented in TPM for metagenome), *Azobacteroidaceae* (9%), *Rikenellaceae* (16%), *UBA4181* (10.1%), *Tannerellaceae* (6.1%), and *UBA932* (3.9%). The same pattern was followed in expressing these genes (Fig. S2a and b).

GH abundance was lower in *G. connexa* (259). Approximately 67.3% (presented in TPM) of the secreted GHs were encoded in *Proteobacteria*, followed by *Bacteroidota* (12.4%), *Firmicutes* (9.4%), and *Actinobacteriota* (9.9%) (Fig. 3c). The same trend was seen in the expression of these GHs (215), with *Proteobacteria* accounting for 62% of all GHs (Fig. 3d; Table S12). The secreted GHs from the MAGs assigned to *Proteobacteria* (142) had the highest capacity to degrade fungal cell-wall (36%; presented in TPM), bacterial cell-wall (21%), hemicellulose (13%), and starch (13.3%). *Bacteroidota* also possessed the same capability. The same pattern was also observed in the expressed GHs. The hydrolytic activities of *Proteobacteria* stemmed from the families of *Enterobacteriaceae* (11.2%; presented in TPM for metagenome), *Sphingomonadaceae* (22%), *Rhizobiaceae* (13%), *Microbacteriaceae* (10%), and *Aeromonadaceae* (6.6%) (Fig. S2c). The *Bacteroidota* family, *Dysgonomonadaceae*, also possessed a high GH abundance (9.2%). The same pattern was followed in expressing these genes (Fig. S2d).

**Fig. 4:**
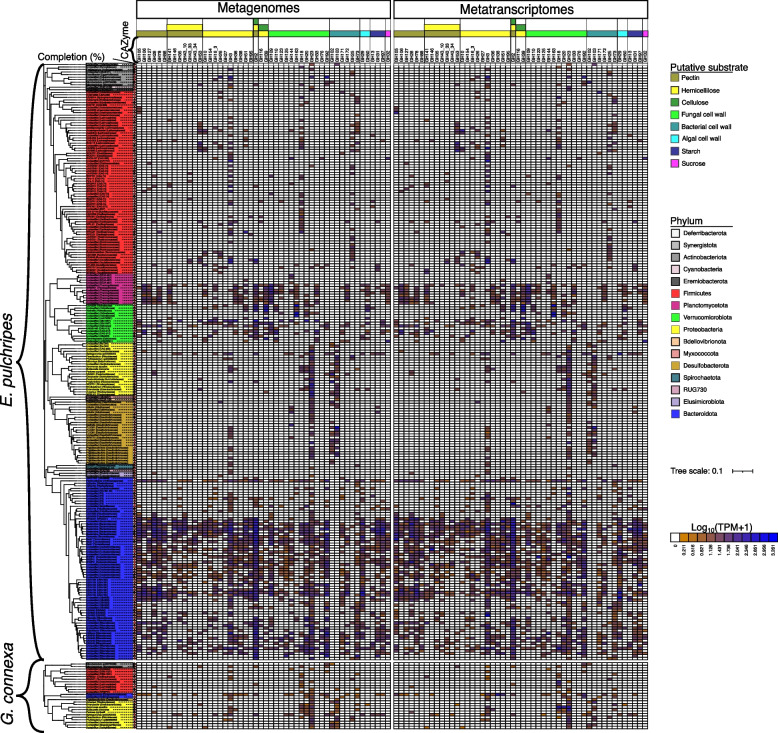
Glycoside hydrolase families and their taxonomic origin. The heatmap shows the relative abundance of the top 50 glycoside hydrolases (GHs) with a secretion signal peptide (SSP) from the MAGs and their corresponding transcripts. The GHs were grouped at the family level according to their putative substrates. The colour scale represents the log transformation of TPM +1. The tree was reconstructed using 39 concatenated bacterial single copy gene (ribosomal proteins) from our MAGs

Figure 4 presents the top 50 GHs belonging to various subfamilies and their putative substrate groups. The top five most prevalent glycoside hydrolase families in *E. pulchripes* were GH43, GH13, GH5, GH3, and GH23. The family GH43, with 316 GHs and 26 subfamilies, was the most abundant. The glycoside hydrolase families GH23, GH18, and GH92 for chitin degradation were among the most abundant GHs. Their abundance in the metatranscriptomes was lower but showed a similar trend. Among the most prevalent glycoside hydrolase (GH) families in *G. connexa* were GH23 and GH18, which break down chitin, and GH13, which break down starch. Others were GH3 (hemicellulose) and GH103 (peptidoglycan). Here as well, the metatranscriptomes showed a similar pattern.

The lignin-degrading CAZymes were scarcely present in both millipede species. The auxiliary activities group of CAZymes (AAs), affiliated in part with ligninolytic activity, made up only about 0.18% (14) of the total CAZymes with SSP (Table S12) in *E. pulchripes*. This group comprised ten AA1 families multicopper oxidases (4 *Bacteroidota*, 5 *Proteobacteria*, and 1 *Verrucomicrobiota)*, and one AA3 (cellobiose dehydrogenase; from *Bacteroidota*), one AA5 (galactose oxidase; from *Myxococcota*), one AA10 (lytic chitin monooxygenase; from *Proteobacteria*), and one AA12 (from *Bacteroidota*). In terms of relative abundance (TPM), the majority of the AAs (42.4%) were sourced from Proteobacteria, with Bacteroidota (30.5%), Myxococcota (24.4%), and Verrucomicrobiota (2.7%) contributing to a lesser extent. The corresponding transcripts also followed a similar pattern. In *G. connexa*, we found only seven AAs: six AA1 (5 *Proteobacteria*, 1 *Desulfobacterota*, and 1 *Firmicutes*) and one AA10 from *Proteobacteria*. In terms of relative abundance, the majority of the AA abundance (78%) was attributed to Proteobacteria, with Desulfobacterota (15%) and Firmicutes (8%) following behind in contribution. Once again, a similar pattern was observed for the corresponding transcripts.

### Acetogenesis in the millipede hindguts

**Fig. 5:**
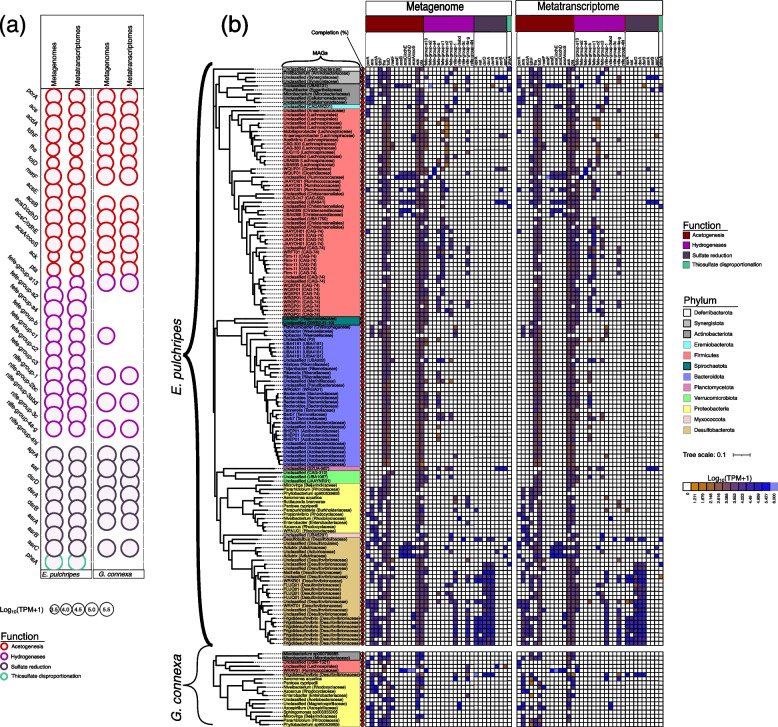
Abundance of gene functions involved in involved acetogenesis, hydrogenases and sulfur cycling pathways in metagenomic and metatranscriptomic libraries and MAGs (**a**) Relative abundance of genes and transcripts for acetogenesis, hydrogen sensing/evolution/bifurcation (hydrogenases) and sulfur cycling in the metagenomic (MG) and metatranscriptomic (MT) contigs from the hindguts of *E*. *Pulchripes* and *G*.* connexa*. Acetogenesis includes heterotrophic acetogenesis via a combination of glycolysis, pyruvate:ferredoxin oxidoreductase (*porA*), Phosphotransacetylase (*pta*) and acetate kinase (*ack*), and reductive acetogenesis via the Wood–Ljungdahl pathway (WLP). Full names of the gene families and their corresponding KEGG IDs are available in Table S13 (**b**) A heatmap showing the abundance of genes and transcripts in each MAG with at least four acetogenic genes or one of the sulfate-reduction genes. The tree was reconstructed using 39 concatenated bacterial single copy gene (ribosomal proteins) from the MAGs

Acetogenesis can act as a sink for excess hydrogen produced during fermentation. We analyzed the community acetogenesis in the assembled reads and found that the key genes for heterotrophic acetogenesis were present and expressed in the libraries of both millipede species (Fig. 5a; Tables S13 and S14). These include pyruvate:ferredoxin oxidoreductase (*porA*), phosphotransacetylase (*pta*), and acetate kinase (*ack*). A pathway involving *porA*, *pta*, *ack*, and the proteins, acetyl-CoA synthetase (ADP-forming, alpha domain) (*acdA*) and acetyl-CoA synthetase (*acs*), plays a vital role in the production and consumption of acetate through the acetate switch [82, 83]. In addition, the essential genes for reductive acetogenesis via the Wood Ljungdahl pathway (*fhs*, *folD*, *metF*, *fdhF*, and *acsABCDE*, *ack*, and *pta*) were also present and expressed in both species, except for methyltransferase (*acsE*), a subunit of acetyl-CoA synthase (*acsABCDE*) which was absent in *G. connexa*. The ability to perform heterotrophic acetogenesis was found in *Proteobacteria*, *Actinobacteria*, *Desulfobacterota*, and *Firmicutes* in *E. pulchripes*, *Proteobacteria*, and *Firmicutes* in *G. connexa*. The capacity for reductive acetogenesis was found in *Desulfobacterota*, *Actinobacteria*, and *Firmicutes* from *E. pulchripes*. *Proteobacteria* from *G. connexa* had all the genes for reductive acetogenesis apart from the *acsABCDE* (Fig. S3a and b).

In the MAGs from *E. pulchripes*, the genes for heterotrophic acetogenesis were encoded by a few MAGs belonging to the core phyla (Fig. 5b; Tables S13 and S14). The three critical genes for acetate production (*porA*, *pta*, and *ack*) were possessed and expressed by two Actinobacteriota MAGs, four Firmicutes MAG, four *Desulfobacterota* MAGs, one *Proteobacteria* MAG, and one *Elusimicrobiota* MAG. Additionally, only two novel, unclassified *Desulfovibrionaceae* MAGs (*Desulfobacterota*) encoded and expressed the five genes for the production or consumption of acetate [83]. The Firmicutes MAGs from *G. connexa* also contained the three genes (*porA*, *pta*, and *ack*). However, these genes were not fully expressed in either of the MAGs.

We searched each MAG for the presence of seven enzymes associated with reductive acetogenesis via the Wood-Ljungdahl pathway (WLP). We found that most of the MAGs encoded and expressed *fdhF*, *fhs*, and *folD*, *ack*, *pta*, but the *metF* and *acsABCDE* were present in only a few MAGs (Fig. 5b; Tables S12 and S13). Of those MAGs, twenty in *E. pulchripes* and two in *G. connexa* encoded at least five of the gene subunits, but none of these MAGs contained the complete set of genes. In *E. pulchripes*, these MAGs included Firmicutes (11 MAGs), *Desulfobacterota* (8 MAGs), *Bacteroidota* (1 MAG), and *Actinobacteriota* (2 MAGs). One additional *Adiutrix* MAG and two Firmicutes lacked only the genes *metF* and *acsE*, while another *Adiutrix* MAG was missing the genes *fdhF*, *metF*, and *acsE*. The MAG that encoded at least five of the genes in *G. connexa* belonged to *Actinobacteriota* and *Firmicutes*. In both millipede species, at least five of the genes were also expressed.

### Hydrogen metabolism in the millipede hindguts

Hydrogenases are required in various anaerobic pathways or H_2_ uptake. Here, we also sought the community genes encoding Ni–Fe hydrogenase, Fe hydrogenase, and FeFe hydrogenase. All the hydrogenase genes (14 orthologs) identified in the community metagenomes were expressed in *E. pulchripes*, except for a [Ni–Fe] group 4 hydrogenase (Fig. 3a; Tables S12 and S13). The only subgroups present and expressed in *G. connexa* were one FeFe hydrogenase and three Ni–Fe hydrogenases.

We identified the genes encoding hydrogenases in the MAGs (Fig. 5b; Tables S13 and S14). Numerous MAGs from *E. pulchripes* encoded one or more types of hydrogenases. Groups A1 and A3 [FeFe] hydrogenases, which are common in many fermentative bacteria, were most prevalent in all MAGs, particularly in *Firmicutes*, *Bacteroidota*, *Planctomycetota*, *Desulfobacterota*, and *Verrucomicrobiota*. [FeFe] hydrogenases of Group A2 and A4 were present in a few *Firmicutes* MAGs and Group B in some *Firmicutes* and *Bacteroidota*. The second most abundant [FeFe] hydrogenases were from Group C1 and were found primarily in *Firmicutes*. [NiFe] hydrogenases were detected mostly in *Desulfobacterota* (Group 1 and Group 4) and a few *Firmicutes* (Group 4). In *G. connexa*, only two *Firmicutes* MAGs encoded a [FeFe] Group A1-hydrogenases. Other [FeFe] hydrogenases were absent. [NiFe] hydrogenases from Groups 1, 3 and 4 were found in a few MAGs of *Proteobacteria*, *Firmicutes*, and *Desulfobacterota*. [Fe] hydrogenases, which are restricted to methanogenic archaea, were absent from both millipede species. Many MAGs expressed multiple hydrogenases, including both [FeFe] and [NiFe] hydrogenases, sometimes up to three paralogues.

### Sulfur metabolism in the millipede hindguts

The prospect of sulfate as an alternative hydrogen sink to acetogenesis was assessed by searching the genes involved in dissimilatory sulfate reduction. We examined the occurrence and expression of key genes involved in sulfate reduction in metagenomes, metatranscriptomes, and MAGs. All the genes were present and expressed in metagenomes from both millipede species, except anaerobic sulfite reductase subunit B (*asrB*) and thiosulfate reductase/polysulfide reductase chain A (*phsA*) in *G. connexa* (Fig. 5a; Table S13). Genes encoding sulfate reductase (*dsrAB*) were present and expressed only in 21 out of the 28 *Desulfobacterota* MAGs from *E. pulchripes*; 19 of them possessed and also expressed *dsrD* (Fig. 5b; Table S14). In addition, we found that 10 MAGs possessed and expressed *aprA* and *sat*. However, both *dsr*, *aprA*, and *sat* genes were absent from the three *Adiutricaceae* MAGs, which agrees with previous results [84]. Among the MAGs from *G. connexa*, only *Desulfovibrionaceae* possessed and expressed *dsrABD*. A thiosulfate reductase (*phsA*) gene involved in thiosulfate disproportionation was present and expressed in several MAGs from *E. pulchripes* (*Desulfovibrionaceae*, 3 MAGs; *Firmicutes*, 1 MAG; *Actinobacteriota*, 2 MAGs). The genes for anaerobic sulfite reduction (*asrABC*) were present and expressed in unclassified *Synergistota* (1 MAG) and *Planctomycetota* (1 MAG) from *E. pulchripes*. The *asr* genes were incomplete in the *Firmicutes* (2 MAGs) from *G. connexa*.

### Nitrogen cycling by millipede hindgut bacteria

**Fig. 6:**
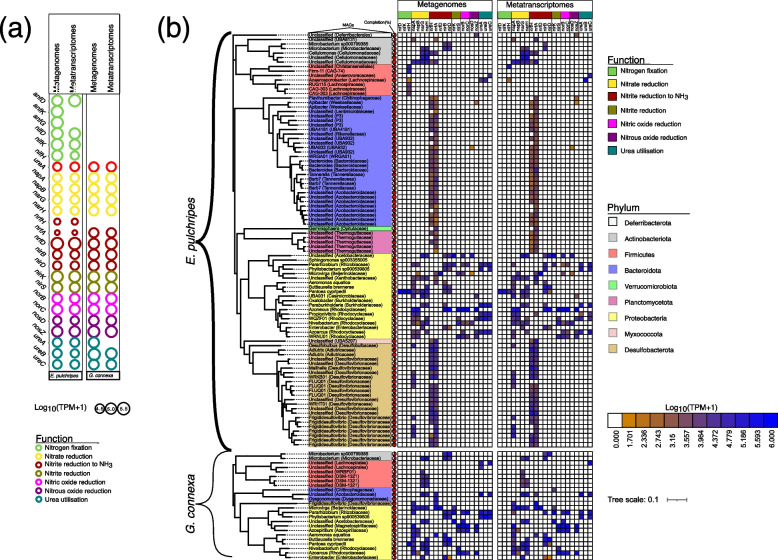
Genes and transcripts involved in nitrogen fixation and nitrogen cycling pathways. **a** Relative abundance of genes and transcripts for nitrogen cycling in the in metagenomic (MG) and metatranscriptomic (MT) contigs from the hindguts of *E*. *Pulchripes* and *G*. *connexa*. Included are the genes involved in nitrogen fixation, nitrite oxidation, nitrate reduction, nitrite reduction to ammonia, nitrite reduction, nitric oxide reduction, nitrous oxide reduction, and urea utilisation. Full names of the gene families and their corresponding KEGG IDs are available in Table S13 (**b**) A heatmap showing the relative abundance of the genes and transcripts in each MAG with at least one of the genes. TPM+1. The tree was reconstructed using 39 concatenated bacterial single copy gene (ribosomal proteins) from our MAGs

As described above, nitrogen fixation and cycling genes can help alleviate the nitrogen demands of detritivores and microbes living in litter. We investigated the presence and expression of key genes involved in nitrogen fixation and cycling in metagenomes, metatranscriptomes, and MAGs (Fig. 6a; Fig S4; Tables S15 and S16). The structural genes of Mo-nitrogenase (*nifDKH*) were present and expressed in the metagenome from *E. pulchripes*. Genes encoding the alternative, Fe–Fe nitrogenase (*anfDGK*), were present, but only *anfD* was expressed. The second alternative, V-Fe nitrogenase (*vnfDKG* genes), was absent. Nitrogenase genes were absent from the metagenome of *G. connexa,* except *nifH*, which was detected in the assembly but was removed due to its short contigs. Genes for aerobic (*amoABC*) or anaerobic ammonium oxidation (*hzoAB*) were absent from the metagenomes. Still, we detected a nitrite oxidoreductase (*nxrAB*) in both millipede species, which may be involved in nitrite oxidation or nitrate reduction. We identified several other genes involved in various forms of nitrogen cycling in both species, including those for nitrate reduction (*napAB* and *narGH*), nitrite reduction to ammonia (*nrfADH* and *nirBD*), nitrite reduction (*nirKS*), nitric oxide reduction (*norBC*), nitrous oxide reduction (*nosZD*), and urea hydrolysis (*ureABC*). However, a portion of the *nrf* gene (*nrfH*) was absent in *G. connexa.* In addition, the urea-hydrolyzing genes (*ureABC*) were present and expressed in both species, except the *ureA* gene, which was not expressed in *G. connexa*.

The *nifDKH* genes in *E. pulchripes* were encoded and expressed by a MAG assigned to *Pantoea cypripedii* (*Proteobacteria*; Fig. 4b and Table S15). The same MAG was also present in *G. connexa*, but the *nifH* gene was not transcribed. In addition, six unclassified *Firmicutes* from *E. pulchripes* (4 *Lachnospiraceae*, 1 *Christensenellales*, and 1 *Oscillospirales* (CAG-74 family)) encoded only the *nifH* gene. The occurrence of active biological nitrogen in *E. pulchripes* but not in *G. connexa* was corroborated using ARA, which showed ethylene accumulation only in *E. pulchripes* (with or without litter; Fig. S5).

Similarly to the case with sulfate, denitrification (nitrate and nitrite reduction) can serve as an alternative respiration pathway for bacteria in the absence of oxygen. For nitrate reduction, the membrane-bound nitrate reductases (*napAB*) were present and expressed in four MAGs assigned to *Rhodocyclaceae* (Proteobacteria) and one assigned to *Cellulomonadaceae* (Firmicutes) in *E. pulchripes* and two *Proteobacteria* MAGs from *G. connexa* (Fig. 4b)*.* The soluble nitrate reductase genes (*narGH*) were encoded and expressed in four MAGs assigned to *Actinobacteriota* and six assigned *Proteobacteria* in *E. pulchripes*. In *G. connexa*, the genes were expressed in MAGs assigned to *Firmicutes* (3), *Bacteroidota* (1), and *Proteobacteria* (4). The second gene, *nxrAB*, identified as the nitrate reductase gene, was found in the same MAGs possessing *narGH.* The nitrite reductase genes (*nirKS*) and nitric oxide reductases (*norBC*) involved in nitrite and nitric oxide reduction through the Nir pathway were found to be encoded and expressed in *Proteobacteria* in both species of millipedes. Our findings showed that the nitrous oxidase accessory protein (*nosD*) was only present and expressed in the MAGs from *Firmicutes* and *Proteobacteria* in *E. pulchripes*. For the nitrous-oxide reductase (*nosZ*) gene, *Proteobacteria* possessed and expressed the gene in both species. Additionally, MAGs assigned to *Deferribacterota* (1) and *Bacteroidota* (2) also expressed the gene in *E. pulchripes*.

Nitrite reduction to ammonia through the Nrf pathway (*nrfAH*) was present and expressed in MAGs from *Desulfobacterota* (20), *Actinobacteriota* (2), *Verrucomicrobiota* (5), one *Myxococcota* (1), and *Bacteroidota* (23). The *nrfAD* genes were only present in one *Proteobacteria* MAG in both species. The *nirBD* genes were encoded and expressed in five *Proteobacteria* MAGs from *E. pulchripes*, four *Proteobacteria*, and *one Actinobacteriota MAGs* from *G. connexa.*

Ureolytic bacteria are often present in environments where urea is constantly produced, such as the millipede gut [16]. Therefore, we also examined the MAGs of both species of millipedes and identified genes that may be involved in using urea (*ureABC*). These urease subunits were only found in *Proteobacteria* and *Firmicutes* in the *E. pulchripes* MAGs. Specifically, the complete set of ureases was encoded by five *Proteobacteria* MAGs in *E. pulchripes* and two *Proteobacteria* MAGs in *G. connexa*. However, none of the three gene subunits was expressed in either species of millipedes. The *ureAC* subunits were only expressed (Fig. 4b).

## Discussion

Although *E. pulchripes* and *G. connexa* were fed the same diet, they differed strongly in the composition and size of their microbiome. The differences in microbial load in the gut and feces of both species have also been reported for other millipede species using classical methods [85, 86]. Our findings using cultivation and ddPCR revealed variations in microbial abundance between the two species, while using amplicon sequencing and metagenomics, we showed differences in community composition. Despite similar sequencing efforts, we obtained roughly nine times more MAGs from *E. pulchripes* species than *G. connexa*. This variation in microbial concentration between even relatively closely related arthropod species has been documented before and could stem from different morphological or physicochemical gut conditions (pH and oxygen availability) [87].

The microbial community composition was in good agreement between all three profiling methods (amplicon sequencing, metagenomics, and metatranscriptomics), providing mutual support for the methods. Notably, however, *Firmicutes* and *Actinobacteriota* were under-represented in our amplicon-based profiling. Comparing the two millipede species, they resembled their taxonomic composition on the phylum level, and many of the genera were shared. However, they differed remarkably in their relative abundances. While seldom tested directly, we assert that the redox state in the gut is one of the main forces shaping the community composition at higher taxonomic levels [also suggested in [87]. Accordingly, the phylum-level composition in *E. pulchripes*, whose gut redox potential is highly negative and hence reducing [29], was dominated by phyla representing many bacteria capable of anaerobic metabolism, such as *Bacteroidota*, *Firmicutes*, and *Verrucomicrobia*. In contrast, the much smaller *G. connexa*, with a typical positive and hence oxidative gut [16], comprised nearly 50% *Proteobacteria*. Similar taxonomic composition, dominated by *Proteobacteria* with low proportions of *Bacteroidota*, is common to many arthropods [87], including other millipedes [88], terrestrial isopods [89, 90], beetles [91, 92], and in many bamboo-feeding Hemiptera, Orthoptera, Lepidoptera, and Coleoptera [11], where the redox conditions in the gut are expected to be positive. Conversely, termites and cockroaches, with typical anoxic guts and active fermentation, typically have lower proportions of *Proteobacteria* and are dominated by *Bacteroidota* and *Firmicutes*, similar to *E. pulchripes* [93–95]. However, three major phyla in termites, with significant importance to their metabolism, namely, *Spirochaetota*, *Fibrobacterota*, and *Elusimicrobiota*, were rare phyla in our datasets. *Spirochaetota* and *Fibrobacterota* are associated with wood-feeding termites and play an important role in cellulose degradation [96, 97]. At the genus level, *Bacteroides* (*Bacteroidota*) dominated in *E. pulchripes.* In contrast, *Dysgonomonas* (*Bacteroidota*) and *Citrobacter* (*Proteobacteria*) dominated in *G. connexa*. Similar to our results, the hindguts of cockroaches harbored mostly representatives of *Bacteroidaceae*, many of which remained unclassified at the genus level [95]. *Dysgonomonas* dominated in dung beetle larvae and pupa [92, 98].

Following taxonomy classification using GTDB-Tk, we identified potentially novel bacteria, primarily assigned to *Firmicutes* and *Bacteroidota*. Only 11% of the MAGs were classified to the species level, while the remaining 88% were not assigned to any known genera. Among the novel MAGs assigned to Firmicutes, only 12 were classified to the family level, and none were assigned to the genus level, except for one MAG assigned to *Holdemania* from the family *Erysipelotrichaceae*, which has only three published genomes in NCBI. We had only two MAGs assigned to the *Erysipelotrichaceae* family. Additionally, MAGs assigned to the families *Butyricicoccaceae, Ruminococcaceae*, and *Acutalibacteraceae* have no published genomes in NCBI. In *Bacteroidota*, we classified 12 MAGs to the family level and 3 MAGs to the genus level. Among these were the genera *Rikenella* and *Tannerella*, with only three published genomes in NCBI. Furthermore, the family *Azobacteroidaceae* has no published genomes in NCBI. Our findings suggest that a substantial number of novel genera or higher taxonomic ranks were detected in these millipede species. The detection of methanogenic archaea in both millipedes was expected. While only *E. pulchripes* is considered CH_4_-emitting, molecular evidence for the presence of methanogenic DNA was also reported for *G. connexa* [30].

The most dominant fungal group, *Ascomycota*, is common in invertebrate gut microbiomes [85, 99], including other millipedes [17]. Since *Ascomycota* are dominant in leaf litter (especially in its early stages of decomposition [100]), they are probably ingested with the leaves and are not residents of the gut microbiome. Rhabditid nematodes, another abundant eukaryote found in *E. pulchripes*, have been reported in the gut of various millipedes [101, 102]. However, their role in the gut microbiome remains unclear. Different protists were also among the dominant eukaryotic groups in both millipede species, though their abundance varied. Protists of the phylum *Ciliophora* (ciliates) are known to host and support symbiotic methanogens in termites thanks to their ability to generate hydrogen [103, 104]. It is therefore likely, though not yet shown, that this is also their role in millipedes. Lastly, *Eccrinales*, a protist order formally considered fungi and grouped together as *Trichomycetes*, are commonly found in many (but not all) millipedes and are considered a regular, non-pathogenic part of the gut microbiome [105]. In our dataset, they were only found as single or double very rare contigs (under 0.2% of the Eukaryotic abundance) and were absent in the metatranscriptome. Therefore, their role in millipedes remains elusive.

Plant material comprises structural components, including cellulose, hemicellulose, pectin, and lignin [106]. In many herbivores, the genomes of individual gut bacteria often encode hundreds of enzymes that help degrade complex plant polysaccharides [6]. Different substrates require different digestive enzymes, which occasionally work in concert [107]. Since polysaccharides typically cannot pass through the cell membrane, we focused only on the CAZymes possessing secretion signal peptides (SSP) that could be released into extracellular space and act on substrates outside the cells [80]. Most CAZymes with SSP were glycoside hydrolases (GHs), the primary enzyme families responsible for polysaccharide degradation [108]. Analysis of GHs with SSP in our MAGs showed that numerous predicted proteins were encoded and expressed. The majority of recent studies on millipedes [24] and other arthropods [14, 109, 110] also revealed significant levels of microbial GHs. However, these studies did not differentiate between GHs with and without signal peptides.

Both *E. pulchripes* and *G. connexa* possessed an abundance of secreted GHs that can degrade pectin (GH28, GH78, GH105, GH106, GH127, and GH88) and hemicellulose (GH2 and GH3), with pectin methylesterases (GH78, GH105, GH106, GH127, and GH88) being particularly prevalent. This could be because of the need to de-esterify homogalacturonan, a common component of plant cell walls [111]. The GH2 enzyme has multiple functions, such as *β-galactosidases*, *β-glucuronidases*, *β-mannosidases*, and *exo-β-glucosaminidases*, which assist in the degradation of hemicellulose. Similarly, the GH3 enzyme aids in plant and bacterial cell wall remodeling, cellulosic biomass degradation, energy metabolism, and pathogen defense [112]. Other abundant GHs include those that can degrade fungal cell walls, which comprise chitin, beta-glucans, and glycoproteins [28]. These were the most abundant GHs in *G. connexa* and the third most abundant in *E. pulchripes*. In addition to chitin, millipedes may also obtain macronutrients, including calcium (present as calcium oxalate), from feeding on fungi and thus support their diet [113]. Additionally, we found a high abundance of some GH families responsible for breaking down the carbohydrate backbone of bacterial peptidoglycans (GH73) and algal cell walls (GH29 and GH50). This suggests that leaf litter-colonizing microorganisms (primarily fungi and bacteria, and maybe also algae) are ingested with the food and serve as a carbon source. Both structural compounds and microorganisms are used as carbon and energy sources in some macroarthropods [114].

The secreted GHs were expressed by different taxa, reflecting the overall differences in community composition. In *E. pulchripes*, the dominant phylum was *Bacteroidota*, which had the highest abundance of GHs for complex carbon degradation at the genome-resolved levels. *Bacteroidota* is known for its ability to break down various complex polysaccharides [115] and is the most polysaccharolytic phylum in cockroaches [116]. On the other hand, *Proteobacteria* were the main source of GHs in *G. connexa*, similar to beetles (Coleoptera) [117]. Both *Bacteroidota* and *Proteobacteria* are agents of complex polysaccharide degradation in isopods [89]. There was also a high level of contributions from *Verrucomicrobiota*, *Firmicutes*, *Planctomycetota*, and *Proteobacteria* in *E. pulchripes* and *Bacteroidota* and *Firmicutes* in *G. connexa.* The success of *Bacteroidota* as a major polysaccharide degrader in *E. pulchripes* was linked to families of *Rikenellaceae*, *Bacteroidaceae*, *UBA4181*, *Azobacteroidaceae*, *Tannerellaceae*, and *UBA932*. Meanwhile, the families of *Sphingomonadaceae*, *Aeromonadaceae*, *Enterobacteriaceae*, and *Rhizobiaceae* significantly contributed to the hydrolytic activities of *Proteobacteria* in *G. connexa*. Similar families with such capabilities were present in omnivorous American cockroaches [116]. However, in contrast to past termite studies, we could not identify any contributions from the rare millipede phyla *Spirochaetota* and *Fibrobacterota* [14, 93].

In animals that rely on symbiotic digestion of (ligno)cellulose, the fermentation products of the bacterial symbionts fuel the carbon and energy metabolism of the host [25]. The hydrogen formed in the fermentations is either converted to methane or—in the case of termites—used for reductive acetogenesis [118]. In some large millipede species, *Archispirostreptus gigas* and *Epibolus pulchripes*, acetate, and formate have been shown to accumulate in the gut, indicating bacterial fermentation activities in the digestive tracts [29]. In heterotrophic metabolism, the enzyme pyruvate:ferredoxin oxidoreductase (*porA*) oxidatively decarboxylates pyruvate to form acetyl-CoA and CO_2_ [119]. Two molecules of acetyl-CoA are converted to two acetate molecules through phosphotransacetylase (*pta*) and acetate kinase (*ack*). In our study, we identified heterotrophic metabolism in some phyla from *E. pulchripe*s that possessed and expressed the *porA*||*pta*||*ack* genes. The activity of *porA* has been identified as the sole site of energy conservation in the model acetogen, *Acetobacterium woodii* [118, 119]. However, it is worth noting that while acetate production is often used as a marker for acetogens, it is not definitive proof of acetogenesis, as other bacteria may also produce acetate. To be considered a true acetogen, a bacterium must be able to perform reductive acetogenesis, which involves using the Wood Ljungdahl Pathway (WLP) to convert two molecules of CO_2_ produced by the oxidative decarboxylation of pyruvate into additional acetate [120]. Based on the seven key enzymes of reductive acetogenesis, we identified formate dehydrogenase H (*fdhF*), formate-tetrahydrofolate ligase/formyl tetrahydrofolate synthetase (*fhs*), methenyltetrahydrofolate cyclohydrolase (*folD*), methylenetetrahydrofolate reductase (*metF*), acetyl-CoA synthase (*acsABCDE*), phosphotransacetylase (*pta*), and acetate kinase (*ack*) [121] in our MAGs. In *E. pulchripes*, putative acetogens with near-complete pathways were found in MAGs assigned to *Firmicutes* (*Clostridaceae*) and *Desulfobacterota* (*Adiutricaceae*). The expression of *fdhF* and *acsABCDE* in these two phyla is a strong predictor for reductive acetogenesis. Two or three missing reductive acetogenic genes in *E. pulchripes* could be related to the incompleteness of our MAGs. The complete *acsABCDE* genes were lacking in MAGs from *G. connexa*, and other genes may not be sufficient to suggest that reductive acetogenesis is present in this species.

Some members of the family *Clostridaceae* (*Firmicutes*) are well-studied acetogens [121]. Microbiota studies have identified acetogenic bacteria belonging to *Firmicutes* [122, 123] from termites and particularly *Ruminococcaceae* in the rumen [120]. Similarly, *Adiutricaceae* in the phylum *Desulfobacterota* from termite guts have also been postulated to be putative acetogens [94, 124]. However, similar to our data, Arora et al. also reported a dominant *Adiutricaceae* MAG with an incomplete pathway [124]. In addition, although belonging to the phylum *Desulfobacterota* [108], which includes many sulfate reducers, none of the MAGs classified as *Adiutricaceae* encoded for the *dsrAB* genes required for dissimilatory sulfate reduction. Therefore, these organisms could also be scavenging hydrogen. A similar observation was made in termites [84, 124].

In addition to genes involved in reductive acetogenesis found in the MAGs from *E. pulchripes*, the putative acetogens possessed and expressed one or more [FeFe] or [NiFe] hydrogenase subgroups. These FeFe hydrogenase subgroups [FeFe] in the Group A-C series are used for H_2_-evolution reaction/electron-bifurcation (*fefe-group-a1,3*), H_2_-uptake/electron-bifurcation (*fefe-group-a4*), H_2_-uptake (*fefe-group-b*), and H_2_-sensing (*fefe-group-c1-3*). The [NiFe] hydrogenase group found in the putative acetogens is for H_2_-uptake (*nife-group-1*) and evolution (*nife-group-4a-g*) [125].

Sulfate-reducing bacteria in the gut of millipedes have not been reported, but their consistent presence in the intestinal tract of many arthropods [126, 127] suggests that they may play a role either in the consumption of hydrogen produced by fermenting bacteria (which requires the presence of sulfate) or the production of hydrogen through fermentation [126, 128]. Although sulfate concentrations in millipede guts are most likely minuscule, similar to those in termites [129], the expression of the *sat*, *aprA*, and *dsrABD* genes and [NiFe] hydrogenases of Group 1 that are involved in H_2_ uptake [126] indicate that the MAGs of *Desulfovibrionaceae* possess the ability to reduce sulfate. Another piece of evidence is the expression of the *acdA* and *acs* genes, which shows that *Desulfovibrionaceae* can use acetate to reduce sulfate since acetate is a competitive substrate for sulfate-reducing bacteria [130]. The expression of the *phsA* gene for thiosulfate disproportionation suggests that at least some of the sulfide produced in this process is reoxidized by the same organisms in the microoxic gut periphery [131], which likely provides the same microoxic conditions as in other arthropods [25].

Leaf litter has a notoriously high C:N ratio, and millipedes and their microbiome are likely permanently nitrogen-starved. Several arthropods living on an N-poor diet have been demonstrated to fix atmospheric nitrogen [132]. The presence and expression of Molybdenum-dependent nitrogenases (*nifDHK*) by *Pantoea cypripedii* (*Proteobacteria*) indicate that the gut microbiota of *E. pulchripes* contributes to dinitrogen reduction. Members of the genus *Pantoea* frequently form associations with various hosts, such as insects, plants, and humans, and are well known for their ability to fix nitrogen [133, 134]. The positive results from the ARA experiment demonstrate that biological nitrogen fixation is occurring in *E. pulchripes*.

As in termites, the gut microbiota of millipedes may also contribute to nitrogen metabolism by recycling uric acid or urea, which are waste products of the host [135, 136] or by reducing dietary nitrate [137]. We found that the gut microbiota of both *E. pulchripes* and *G. connexa* expresses genes involved in urea oxidation, denitrification, and dissimilatory nitrate reduction to ammonia (DNRA). Denitrification is an important process in various soil fauna, including earthworms [138] and termites [137]. The most important contributors to these activities in *E. pulchripes* (Spirobolida) and *G. connexa* (Glomeridae) are *Actinobacteria*, *Firmicutes*, and *Proteobacteria*. Assuming that denitrification produces traces of N_2_O, the production of this greenhouse gas may not be restricted to the Glomeridae family, as previously thought [139]. DNRA activities have been documented by stable-isotope analyses in soil-feeding termites [137, 140] and several freshwater insects [140]. Key genes of DNRA are the nitrite reductases *nrfA* and *nirB* [141]. They were expressed by members of *Cellulomonadaceae* (*Actinobacteriota*) from *E. pulchripes* and members of *Proteobacteria* from *E. pulchripes* and *G. connexa*. Likewise, the presence of the *nrfAH* genes has been established in the gut microbiota of termites [124, 142] and aquatic insects [140].

Several MAGs of *Proteobacteria* from *E. pulchripes* and *G. connexa* also expressed ureases (*ureABC*), suggesting they contribute to ammonia production from urea. Urease activity is common in many bacteria from host-associated environments [143, 144]. *Proteobacteria* and *Actinobacteria* with urease activity have been isolated from millipede guts [86, 145].

## Conclusions

The data presented here is a comprehensive chart of the metabolic diversity in two millipede model species; one of Earth’s most important groups of detritivores. We found substantial differences in both abundance and diversity of the gut microbial community between two millipede species that differ in their size, habitat, and gut redox conditions but share the same diet and lifestyle. Many functions encoded by the gut microbiota were present in the MAGs of both species, including the capacity to degrade complex carbohydrates. Lignin-modifying enzymes were very few, but a high expression of genes for chitin degradation indicates that fungal biomass may play an important role in the millipede diet, perhaps exceeding that of plant polymers. Fermentative lineages (*Clostridiales* and *Bacteroidales*) were particularly abundant in the large *E. pulchripes*, but clear evidence for reductive acetogenesis was lacking. Instead, we found strong evidence for hydrogenotrophy, nitrogen recycling, and diazotrophy. The results should serve as a roadmap for further studies to test these hypotheses regarding the trophic role of millipedes.

### Supplementary Information

**Additional file 1:** **Fig. S1.** The relative abundance of eukaryotes in assembled metagenomic and metatranscriptomic reads from hindguts of *E. pulchripes* and *G. connexa*. (a) Abundance of fungi in (a) metagenome and (b) metatranscriptome. Abundance of algae and protists in (a) metagenome and (b) metatranscriptome. The paired-end reads of both library types were mapped to the genes/contigs to obtain the coverage and calculate the relative abundance in Transcript Per Million (TPM). The mean TPM was calculated from the three replicate samples and aggregated for each taxonomic level. **Fig. S2.** Relative abundance of glycoside hydrolases (GHs) with signal peptides in metagenome-assembled genomes (MAGs) and their corresponding transcripts. The GHs were grouped at the family level and according to their putative substrates (top of each chord) and the top 3 taxa (at the family level) contributing the GHs (bottom of each chord). Chord (a) displays the contribution of GHs from different families in metagenomes, while chord (b) shows its corresponding GH transcripts from the hindgut of *E. pulchripes*. Chord (c) shows the abundance of GHs at the family level in metagenomes, while chord (d) displays its corresponding GH transcripts from the hindgut of *G. connexa*. The pair-end reads of both library types were mapped to the genes to get the coverage and calculate the relative abundance in Transcript Per Million (TPM). The mean TPM was calculated from the three replicate samples and summed for each taxonomic level. **Fig. S3.** Relative abundance and taxonomic distribution of genes involved in acetogenesis, hydrogen sensing/evolution/bifurcation (hydrogenases) and sulfur cycling in the metagenomic (MG) and metatranscriptomic (MT) contigs from the hindguts of *E. Pulchripes* and *G. connexa.* (a) Boxplots showing the relative abundance of the bacterial genes for a function within a phylum. (b) Taxonomic distribution of genes and transcripts at the phylum level. The pair-end reads from metagenomes and metatranscriptomes were mapped to all the genes to get their coverages and averaged to estimate their relative abundance in transcript per kilobase million (TPM). **Fig. S4.** Relative abundance and taxonomic distribution of genes involved in nitrogen fixation and recycling, and their corresponding transcripts. (a) Boxplots showing the relative abundance of the genes for a function within a phylum. (b) Taxonomic distribution of the genes and transcripts at the phylum level. The pair-end reads from metagenomes and metatranscriptomes were mapped to all the genes to get their coverages and averaged to estimate their relative abundance in transcript per kilobase million (TPM). **Fig. S5.** Functional assay for the activity of the N_2_-fixing nitrogenase enzyme in the reduction of acetylene to ethylene in *E. pulchripes*.

**Additional file 2:** **Table S1.** Mean values for colony counts and 16S rRNA copies. **Table S2.** Read counts and taxonomic classification of 16S rRNA amplicon sequence. **Table S3.** Sample information for metagenomic and metatranscriptomic sequencing. **Table S4.** Contig stats for de novo co-assembled reads from E. pulchripes. **Table S5.** Removal of potential eukaryotic and unknown contigs with Whokaryote. **Table S6.** Contig stat for de novo co-assembled reads from E. pulchripes and G. connexa. **Table S7.** Taxonomic classification of MAGs with GTDB-Tk. **Table S8.** Taxonomic classification of MAGs from E. pulchripes and G . connexa. **Table S9.** MAG coverage in quality-filtered metagenomic pair-end reads **Table S10.** Distributions of all CAZymes with or without signal peptides in bacterial MAGs from E. pulchripes and G. connexa. **Table S11.** Grouping of glycoside hydrolases at the family level. **Table S12.** Annotated Carbohydrate-active enzyme (CAZymes) from MAGs using dbCAN2 meta web server. **Table S13.** Community gene annotation of the metagenome and metatranscriptome samples (prokaryotic co-assemblies). **Table 14.** Community relative abundance of genes involved in acetogenesis, hydrogenases and sulfur cycling for E. pulchripes and G. connexa. **Table S15.** Community gene annotation of the metagenome and metatranscriptome samples (prokaryotic co-assemblies). **Table S16.** Community relative abundance of genes involved in nitrogen cycling. **Table S17.** NCBI BioProject PRJNA948469.

## Data Availability

The shotgun metagenomes, metatranscriptomes, and short-read amplicon sequencing data were deposited under the NCBI BioProject PRJNA948469 (see Table S17). For reproducibility, reusability, and transparency, we have also made available the FASTA file for the co-assembled metagenomes from *Epibolus pulchripes* and *Glomeris connexa* and the anvi'o merged profile database (doi: 10.6084/m9.figshare.22336732). The FASTA file and anvi'o merged profile database for *G. connexa* can be found at doi: 10.6084/m9.figshare.22339522and for *E. pulchripes* at doi: 10.6084/m9.figshare.22337500. The scripts used in this study are available on GitHub (https://github.com/julipeale2001/Millipede-gut-microbiome-metaomics-analysis).
