# Supplementary material for: Functional similarity, despite taxonomical divergence in the millipede gut microbiota, points to a common trophic strategy
Source: Microbiome. 2024 Jan 29;12:16. doi: 10.1186/s40168-023-01731-7 (PMC10823672; doi:10.1186/s40168-023-01731-7)
Supplement: Supplementary file 2 — Additional file 1: Fig. S1. The relative abundance of eukaryotes in assembled metagenomic and metatranscriptomic reads from hindguts of E. pulchripes and G. connexa. (a) Abundance of fungi in (a) metagenome and (b) metatranscriptome. Abundance of algae and protists in (a) metagenome and (b) metatranscriptome. The paired-end reads of both library types were mapped to the genes/contigs to obtain the coverage and calculate the relative abundance in Transcript Per Million (TPM). The mean TPM was calculated from the three replicate samples and aggregated for each taxonomic level. Fig. S2. Relative abundance of glycoside hydrolases (GHs) with signal peptides in metagenome-assembled genomes (MAGs) and their corresponding transcripts. The GHs were grouped at the family level and according to their putative substrates (top of each chord) and the top 3 taxa (at the family level) contributing the GHs (bottom of each chord). Chord (a) displays the contribution of GHs from different families in metagenomes, while chord (b) shows its corresponding GH transcripts from the hindgut of E. pulchripes. Chord (c) shows the abundance of GHs at the family level in metagenomes, while chord (d) displays its corresponding GH transcripts from the hindgut of G. connexa. The pair-end reads of both library types were mapped to the genes to get the coverage and calculate the relative abundance in Transcript Per Million (TPM). The mean TPM was calculated from the three replicate samples and summed for each taxonomic level. Fig. S3. Relative abundance and taxonomic distribution of genes involved in acetogenesis, hydrogen sensing/evolution/bifurcation (hydrogenases) and sulfur cycling in the metagenomic (MG) and metatranscriptomic (MT) contigs from the hindguts of E. Pulchripes and G. connexa. (a) Boxplots showing the relative abundance of the bacterial genes for a function within a phylum. (b) Taxonomic distribution of genes and transcripts at the phylum level. The pair-end reads from metagenomes a [file 40168_2023_1731_MOESM1_ESM.docx]

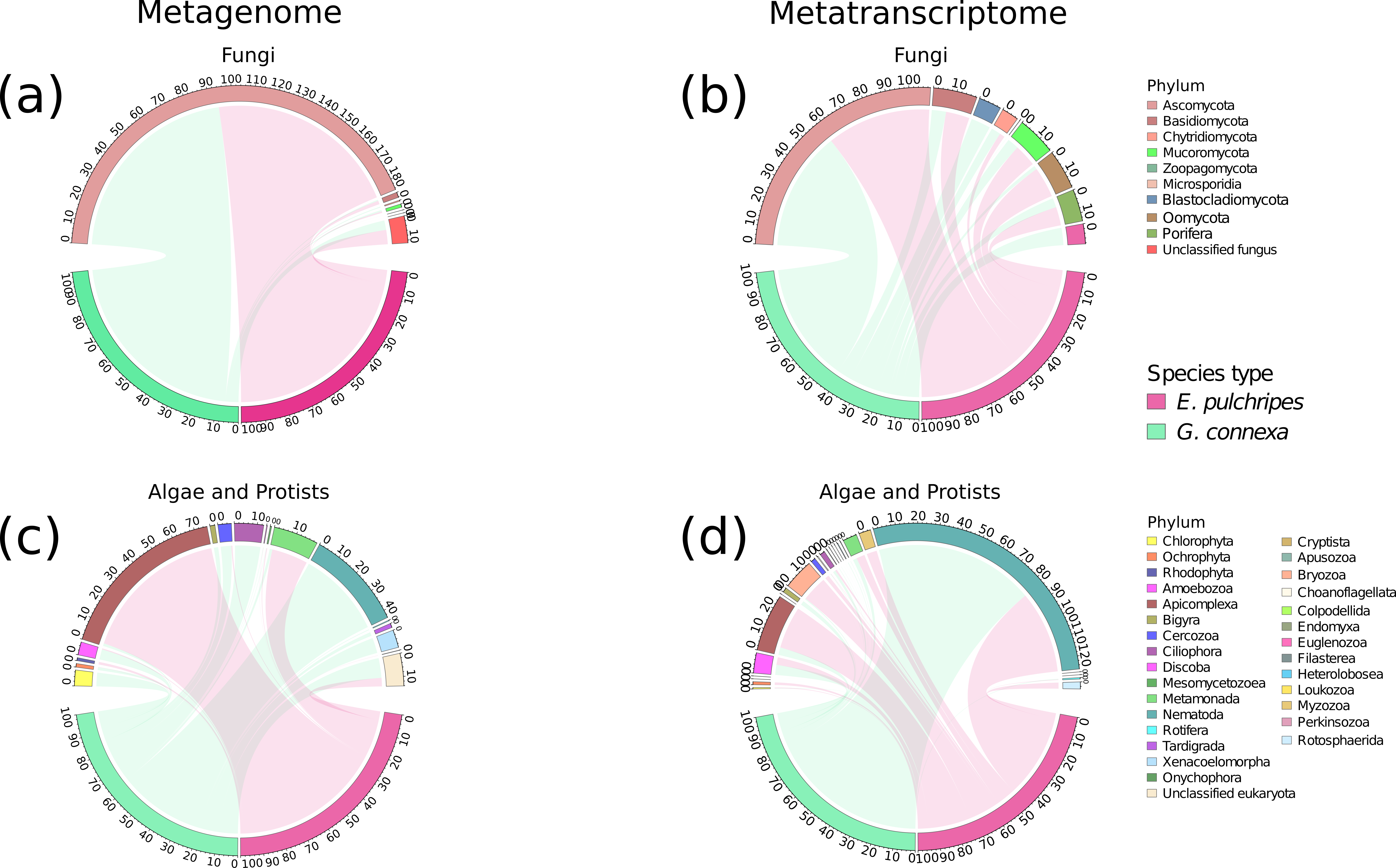


**Fig. S1. The relative abundance of eukaryotes in assembled metagenomic and metatranscriptomic reads from hindguts of *E. pulchripes* and *G. connexa***. (a) Abundance of fungi in (a) metagenome and (b) metatranscriptome. Abundance of algae and protists in (a) metagenome and (b) metatranscriptome. The paired-end reads of both library types were mapped to the genes/contigs to obtain the coverage and calculate the relative abundance in Transcript Per Million (TPM). The mean TPM was calculated from the three replicate samples and aggregated for each taxonomic level.


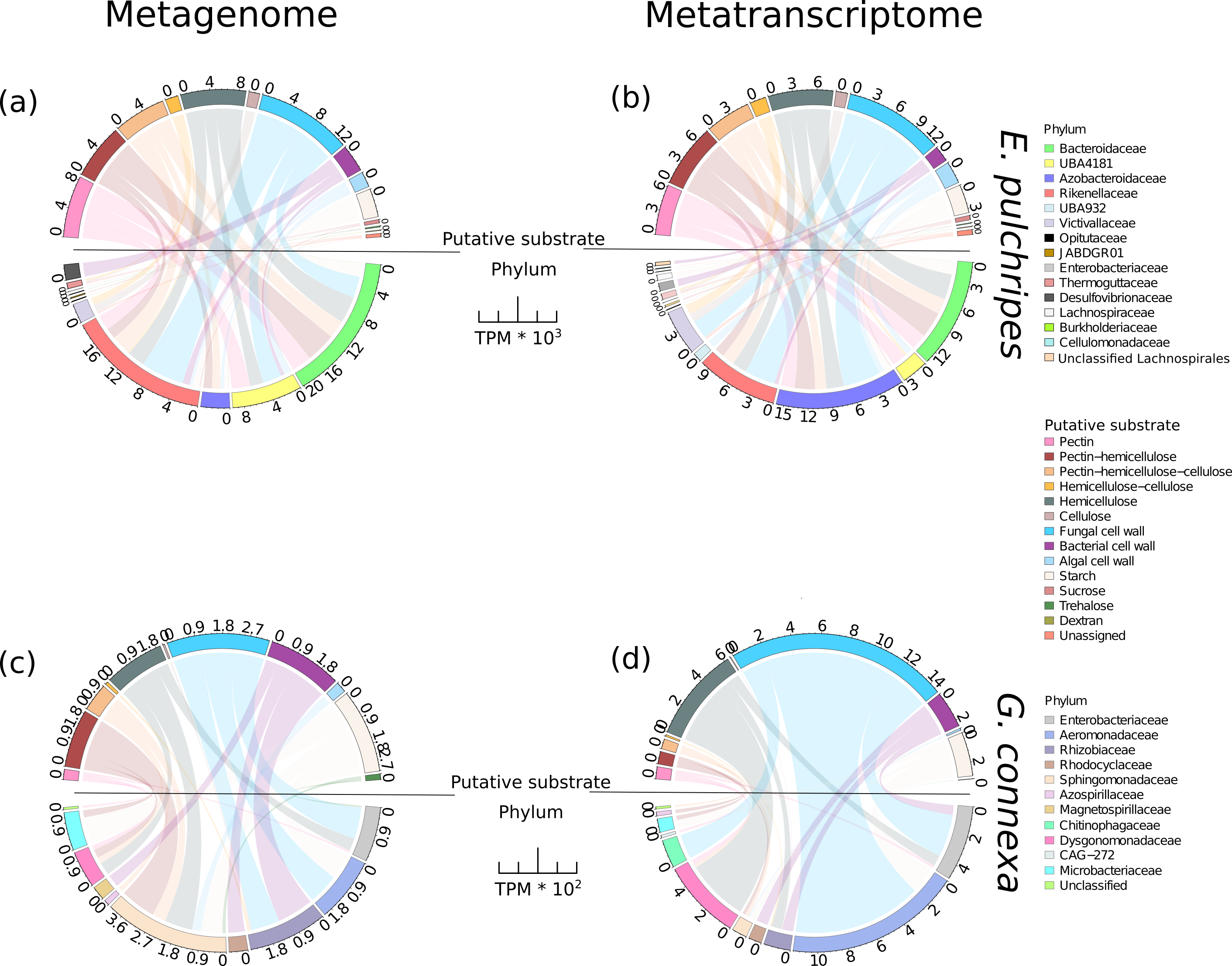


**Fig. S2. Relative abundance of glycoside hydrolases (GHs) with signal peptides in metagenome-assembled genomes (MAGs) and their corresponding transcripts.** The GHs were grouped at the family level and according to their putative substrates (top of each chord) and the top 3 taxa (at the family level) contributing the GHs (bottom of each chord). Chord **(a)** displays the contribution of GHs from different families in metagenomes, while chord **(b)** shows its corresponding GH transcripts from the hindgut of *E. pulchripes*. Chord **(c)** shows the abundance of GHs at the family level in metagenomes, while chord **(d)** displays its corresponding GH transcripts from the hindgut of *G. connexa*. The pair-end reads of both library types were mapped to the genes to get the coverage and calculate the relative abundance in Transcript Per Million (TPM). The mean TPM was calculated from the three replicate samples and summed for each taxonomic level.


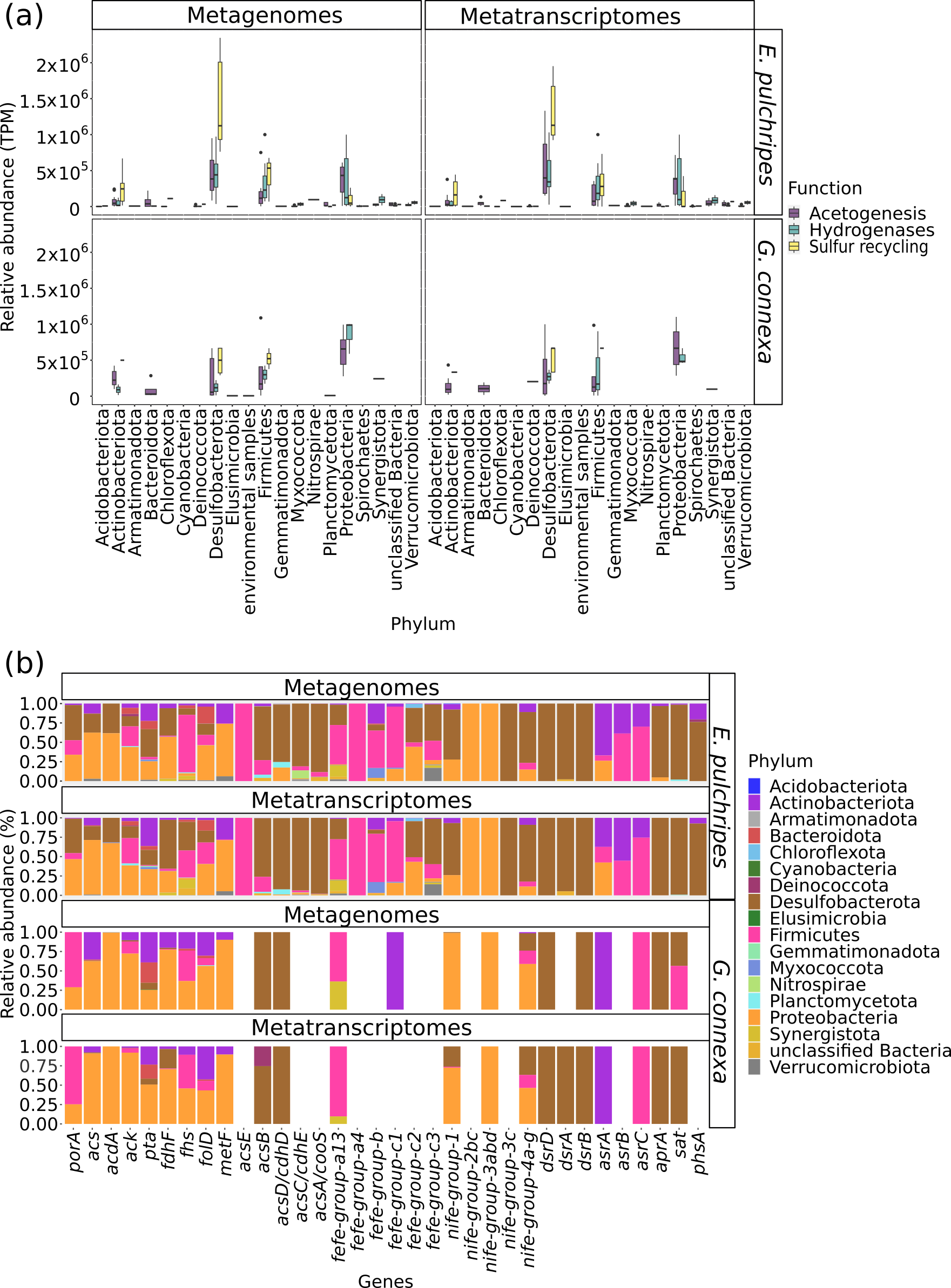


**Fig. S3. Relative abundance and taxonomic distribution of genes involved in acetogenesis, hydrogen sensing/evolution/bifurcation (hydrogenases) and sulfur cycling in the metagenomic (MG) and metatranscriptomic (MT) contigs from the hindguts of *E. Pulchripes* and *G. connexa.* (a)** Boxplots showing the relative abundance of the bacterial genes for a function within a phylum. **(b)** Taxonomic distribution of genes and transcripts at the phylum level. The pair-end reads from metagenomes and metatranscriptomes were mapped to all the genes to get their coverages and averaged to estimate their relative abundance in transcript per kilobase million (TPM).


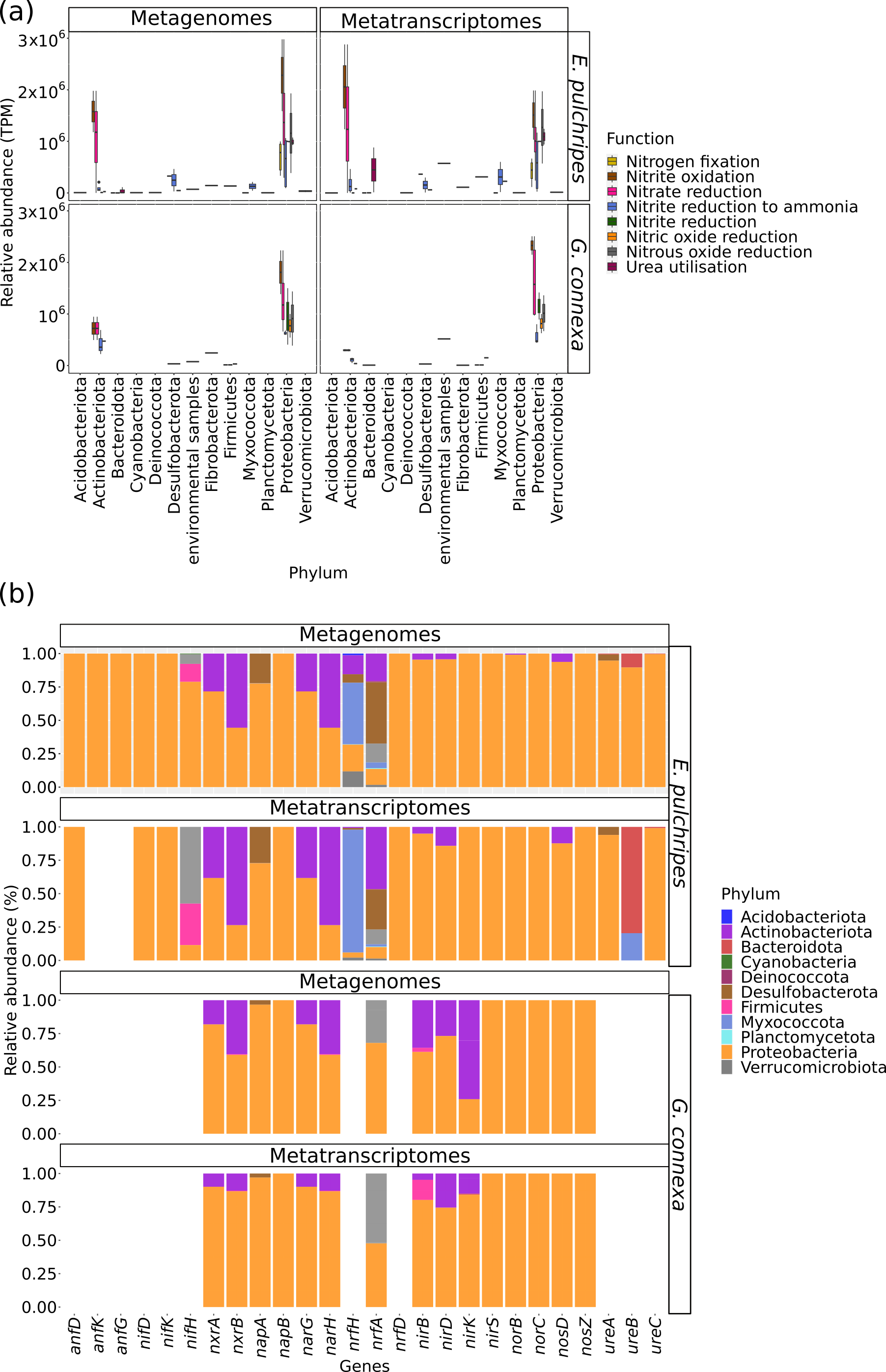


**Fig. S4. Relative abundance and taxonomic distribution of genes involved in nitrogen fixation and recycling, and their corresponding transcripts.** **(a)** Boxplots showing the relative abundance of the genes for a function within a phylum. **(b)** Taxonomic distribution of the genes and transcripts at the phylum level. The pair-end reads from metagenomes and metatranscriptomes were mapped to all the genes to get their coverages and averaged to estimate their relative abundance in transcript per kilobase million (TPM).


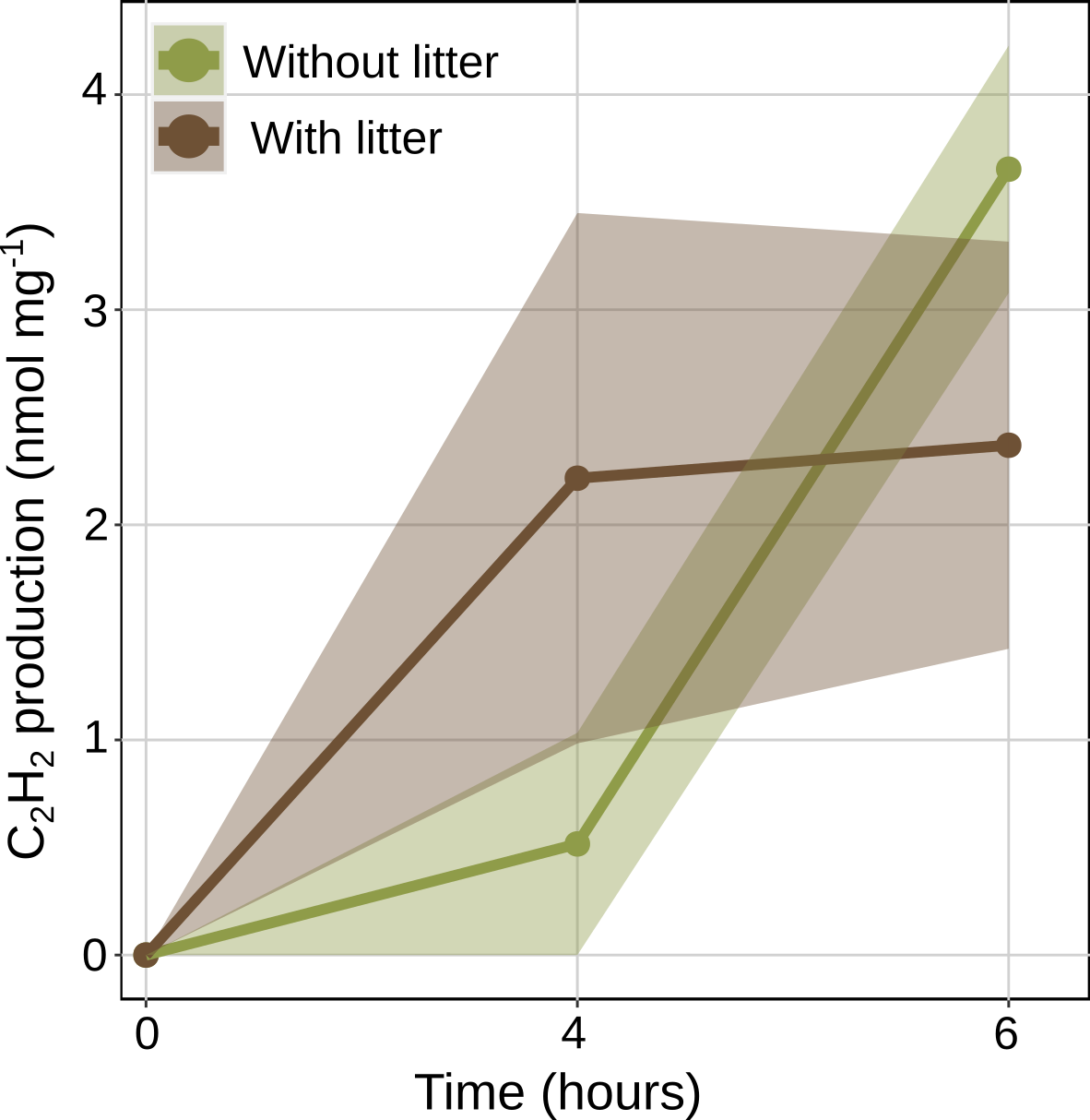


**Fig. S5. Functional assay for the activity of the N_2_-fixing nitrogenase enzyme in the reduction of acetylene to ethylene in *E. pulchripes*.**
